# Mitigating site effects in covariance for machine learning in neuroimaging data

**DOI:** 10.1002/hbm.25688

**Published:** 2021-12-14

**Authors:** Andrew A. Chen, Joanne C. Beer, Nicholas J. Tustison, Philip A. Cook, Russell T. Shinohara, Haochang Shou, The Alzheimer's Disease Neuroimaging Initiative

**Affiliations:** ^1^ Penn Statistics in Imaging and Visualization Center, Department of Biostatistics, Epidemiology, and Informatics University of Pennsylvania Philadelphia Pennsylvania USA; ^2^ Center for Biomedical Image Computing and Analytics University of Pennsylvania Philadelphia Pennsylvania USA; ^3^ Department of Radiology and Medical Imaging University of Virginia Charlottesville Virginia USA; ^4^ Department of Radiology University of Pennsylvania Philadelphia Pennsylvania USA

**Keywords:** ComBat, cortical thickness, covariance, harmonization, multi‐site analysis, site effect

## Abstract

To acquire larger samples for answering complex questions in neuroscience, researchers have increasingly turned to multi‐site neuroimaging studies. However, these studies are hindered by differences in images acquired across multiple sites. These effects have been shown to bias comparison between sites, mask biologically meaningful associations, and even introduce spurious associations. To address this, the field has focused on harmonizing data by removing site‐related effects in the mean and variance of measurements. Contemporaneously with the increase in popularity of multi‐center imaging, the use of machine learning (ML) in neuroimaging has also become commonplace. These approaches have been shown to provide improved sensitivity, specificity, and power due to their modeling the joint relationship across measurements in the brain. In this work, we demonstrate that methods for removing site effects in mean and variance may not be sufficient for ML. This stems from the fact that such methods fail to address how correlations between measurements can vary across sites. Data from the Alzheimer's Disease Neuroimaging Initiative is used to show that considerable differences in covariance exist across sites and that popular harmonization techniques do not address this issue. We then propose a novel harmonization method called Correcting Covariance Batch Effects (CovBat) that removes site effects in mean, variance, and covariance. We apply CovBat and show that within‐site correlation matrices are successfully harmonized. Furthermore, we find that ML methods are unable to distinguish scanner manufacturer after our proposed harmonization is applied, and that the CovBat‐harmonized data retain accurate prediction of disease group.

## Graphical abstract

Multi‐site neuroimaging studies are hindered by differences in images acquired across multiple sites, often referred to as site effects. In this work, we demonstrate that methods for removing site effects in mean and variance may not be sufficient for machine learning. After applying our proposed harmonization method CovBat, we find that machine learning methods are unable to distinguish scanner manufacturer after our proposed harmonization is applied, and that the CovBat‐harmonized data retain accurate prediction of disease group.

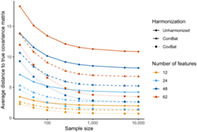

## 1 INTRODUCTION

The need for larger samples in human subjects research have led to a growing number of multi‐site studies that aggregate data across multiple locations. This trend is especially prevalent in neuroimaging research where the reliability and generalizabilty of findings from the conventional single‐site studies are often limited by the ability to recruit and study sufficiently large and representative samples from the population. Many consortia have been formed to address such issues (Mueller et al., 2005; Sudlow et al., 2015; Trivedi et al., 2016; Van Essen et al., 2013). The larger samples obtained through these efforts promote greater power to detect significant associations as well as better generalizability of results. However, these study designs also introduce heterogeneity in acquisition and processing that, if not appropriately addressed, may impact study findings.

Several researchers have determined that variability driven by site differences, often called site effects, reduce the reliability of derived measurements, and can introduce bias. Neuroimaging measurements have been repeatedly shown to be affected by scanner manufacturer, model, magnetic field strength, head coil, voxel size, and acquisition parameters (Han et al., 2006; Kruggel, Jessica, Tugan Muftuler, & Initiative, 2010; Reig et al., 2009; Wonderlick et al., 2009). Yet even in scanners of the exact same model and manufacturer, differences still exist for certain neuroimaging biomarkers (Takao, Hayashi, & Ohtomo, 2011).

Until recently, neuroimaging analyses primarily involved mass univariate testing which treats features as independent and does not leverage covariance between features. Under this paradigm, the impact of site effects is through changes in the mean and variance of measurements. Increasingly, researchers have used sets of neuroimaging features as inputs into prediction algorithms or state‐of‐the‐art machine learning (ML) methods. This approach has become a powerful tool for leveraging both functional and structural neuroimaging for research into pain (Smith, López‐Solà, McMahon, Pedler, & Sterling, 2017; Wager et al., 2013), neural representations (Haxby, Connolly, & Swaroop Guntupalli, 2014), and psychiatric illnesses (Koutsouleris et al., 2014). One of the major benefits of ML is that it leverages the joint distribution and correlation structure among multivariate brain features in order to better characterize a phenotype of interest (Gregorutti, Michel, & Saint‐Pierre, 2017; O'Toole et al., 2007). As a result, site effects on the covariance of measurements are likely to impact findings substantially. In fact, a recent investigation showed that an ML algorithm was able to detect scanner with high accuracy and that the detection of sex depended heavily on the scanners included in the training and test data (Glocker, Robinson, Castro, Dou, & Konukoglu, 2019).

The major statistical harmonization techniques employed in neuroimaging have generally corrected for differences across sites in mean and variance, but not covariance (Fortin et al., 2018; Fortin, Sweeney, Muschelli, Crainiceanu, & Shinohara, 2016; Rao, Monteiro, & Mourao‐Miranda, 2017; Yamashita et al., 2019). Increasingly, the ComBat model (Johnson, Cheng, & Rabinovic, 2007) has become a popular harmonization technique in neuroimaging and has been successfully applied to structural and functional measures (Bartlett et al., 2018; Fortin et al., 2017, 2018; Marek et al., 2019; Yu et al., 2018). However, this model does not address potential site effects in covariance.

Recently, another stream of data‐driven harmonization methods have aimed to apply generative adversarial networks (GANs) or distance‐based methods to unify distributions of measurements across sites. However, the GAN‐based harmonization methods have only been tested for harmonization of images and lack options to retain clinical associations of interest (Gao, Liu, Wang, Shi, & Jinhua, 2019; Nguyen, Morris, Harris, Korgoankar, & Ramos, 2018; Zhong et al., 2020). A recent distance‐based method is applicable to derived measurements and has been tested in classification of Alzheimer's disease (AD) using support vector machines (Zhou et al., 2018). However, the method in (Zhou et al., 2018) has not been tested for detection of site via ML and also requires several conditions which may not hold in studies with sufficiently heterogeneous sites or major differences in subject demographics across sites.

In this article, we examine whether site effects influence ML results. In particular, we study the cortical thickness measurements derived from images acquired by the Alzheimer's Disease Neuroimaging Initiative (ADNI) and demonstrate the existence of site effects in covariance of structural imaging measures. We then propose a novel harmonization method called Correcting Covariance Batch Effects (CovBat) that removes site effects in mean, variance, and covariance. We apply CovBat and show that within‐site correlation matrices are successfully harmonized. Furthermore, we find that ML methods are unable to detect Siemens scanners after our proposed harmonization is applied, and that the CovBat‐harmonized data retain accurate prediction of disease group. We also assess the performance of the proposed method in simulated data, and again find that the method mitigates site effects and maintains detection of meaningful associations. Our results demonstrate the need to consider covariance in harmonization methods, and suggest a novel procedure that can be applied to better harmonize data from multi‐site imaging studies.

## 2 MATERIALS AND METHODS

### 2.1 ADNI dataset

The data for this article consist of baseline scans from ADNI (http://adni.loni.usc.edu/ which are processed using the ANTs longitudinal single‐subject template pipeline (Tustison et al., 2019) with code available on GitHub (https://github.com/ntustison/CrossLong). Informed consent was obtained by all participants in the ADNI study. Institutional review boards approved the study at all of the contributing institutions.

We briefly summarize the steps involved. First, we download raw T1‐weighted images from the ADNI‐1 database, which were acquired using MPRAGE for Siemens and Philips scanners and a works‐in‐progress version of MPRAGE on GE scanners (Jack et al., 2010). We choose ADNI‐1 to highlight a severe site effect situation driven by greater variability in scanner properties, including magnetic field strength, which are standardized in later ADNI releases. For each subject, we estimate a single‐subject template from all the image timepoints. After rigid spatial normalization to this single‐subject template, each normalized timepoint image is then processed using the single image cortical thickness pipeline consisting of brain extraction (B. Avants, Klein, Tustison, Woo, & Gee, 2010), denoising (Manjón, Coupé, Luis, Louis Collins, & Robles, 2010), N4 bias correction (N. J. Tustison et al., 2010), Atropos *n*‐tissue segmentation (B. B. Avants, Tustison, Wu, Cook, & Gee, 2011), and registration‐based cortical thickness estimation (Das, Avants, Grossman, & Gee, 2009). For our analyses, we use the 62 baseline cortical thickness values as defined by the Desikan–Killiany–Tourville atlas (Klein & Tourville, 2012). The sample covariance matrix for these cortical thicknesses in the largest site is shown with labels in Figure S1.

We define site based on information contained within the Digital Imaging and Communications in Medicine (DICOM) headers for each scan. Specifically, subjects are considered to be acquired at the same site if they share the scanner location, scanner manufacturer, scanner model, head coil, and magnetic field strength. In total, this definition yields 142 distinct sites of which 78 had less than three subjects and were removed from analyses. The final sample consists of 505 subjects across 64 sites, with 213 subjects imaged on scanners manufactured by Siemens, 70 by Philips, and 222 by GE. The sample has a mean age of 75.3 (SD 6.70) and is comprised of 278 (55%) males, 115 (22.8%) AD patients, 239 (47.3%) late mild cognitive impairment (LMCI), and 151 (29.9%) cognitively normal (CN) individuals.

The ADNI sample demographics are considerably different across sites, which precludes application of certain harmonization methods. For example, (Zhou et al., 2018) relies on “nontrivial overlap” of the potential confounders across sites and proposed a subsampling approach that performs distributional shifts on subsamples of data matched by the discrete stratum of the confounders. Given that our data are sufficiently heterogeneous, it is challenging to form bins matched by age, sex, and diagnosis status to ensure that each site has at least one individual in each bin. This prevents protection of age effects in applying the harmonization method proposed by (Zhou et al., 2018).

### 2.2 Combatting batch effects

We first review ComBat (Fortin et al., 2017, 2018; Johnson et al., 2007) for harmonization of neuroimaging measures. ComBat seeks to remove the mean and variance site effects of the data in an empirical Bayes framework. Let $y_{\mathrm{ij}}={\left(y_{\mathrm{ij}1},y_{\mathrm{ij}2},\ldots ,y_{\mathrm{ijp}}\right)}^{T}$, i=1,2,…,M, $j=1,2,\ldots ,n_{i}$ denote the p×1 vectors of observed data where i indexes site, j indexes subjects within sites, $n_{i}$ is the number of subjects acquired on site i, and p is the number of features. Our goal is to harmonize these vectors across the M sites. ComBat assumes that the features indexed by v follow

$$y_{\mathrm{ijv}}=\alpha_{v}+x_{\mathrm{ij}}^{T}\beta_{v}+\gamma_{\mathrm{iv}}+\delta_{\mathrm{iv}}e_{\mathrm{ijv}}$$

where $\alpha_{v}$ is the intercept, $x_{\mathrm{ij}}$ is the vector of covariates, $\beta_{v}$ is the vector of regression coefficients, $\gamma_{\mathrm{iv}}$ is the mean site effect, and $\delta_{\mathrm{iv}}$ is the variance site effect. The errors $e_{\mathrm{ijv}}$ are assumed to independently follow $e_{\mathrm{ijv}}∼N\left(0,\sigma_{v}^{2}\right)$. ComBat first finds least‐squares estimates ${\hat{\alpha }}_{v}$ and ${\hat{\beta }}_{v}$ for each feature. To estimate the site effects using empirical Bayes, ComBat assumes that the $\gamma_{\mathrm{iv}}$ follow independent normal distributions and the $\delta_{\mathrm{iv}}$ follow independent inverse gamma distributions. The hyperparameters are then estimated via method of moments using data across all features. The empirical Bayes point estimates $\gamma_{\mathrm{iv}}^{*}$ and $\delta_{\mathrm{iv}}^{*}$ are then obtained as the means of the posterior distributions. Finally, ComBat residualizes with respect to these estimates to obtain harmonized data

$$y_{\mathrm{ijv}}^{\text{ComBat}}=\frac{y_{\mathrm{ijv}}-{\hat{\alpha }}_{v}-x_{\mathrm{ij}}^{T}{\hat{\beta }}_{v}-\gamma_{\mathrm{iv}}^{*}}{\delta_{\mathrm{iv}}^{*}}+{\hat{\alpha }}_{v}+x_{\mathrm{ij}}^{T}{\hat{\beta }}_{v}$$

### 2.3 Correcting covariance batch effects

To address potential covariance site effects, we build on the existing ComBat framework. We again assume that the features follow

$$y_{\mathrm{ijv}}=\alpha_{v}+x_{\mathrm{ij}}^{T}\beta_{v}+\gamma_{\mathrm{iv}}+\delta_{\mathrm{iv}}e_{\mathrm{ijv}}$$

However, the error vectors $e_{\mathrm{ij}}={\left(e_{\mathrm{ij}1},e_{\mathrm{ij}2},\ldots ,e_{\mathrm{ijp}}\right)}^{T}∼N\left(0,\sum_{i}\right)$ may be spatially correlated and differ in covariance across site. Analogous to how ComBat modifies observations to bring each within‐site variance to the pooled variance across sites, our proposed method modifies principal component (PC) scores to shift each within‐site covariance to the pooled covariance structure. We achieve this by approximating within‐site covariance structures using the PCs and PC scores obtained across all observations. We propose the CovBat algorithm, which accounts for the joint distribution of ComBat‐adjusted observations as follows:

*Step 1*. We first perform ComBat to remove the mean and variance shifts in the marginal distributions of the cortical thickness measures. Then, we additionally residualize with respect to the intercept and covariates to obtain ComBat‐adjusted residuals denoted $e_{\mathrm{ij}}^{\text{ComBat}}={\left(e_{\mathrm{ij}1}^{\text{ComBat}},e_{\mathrm{ij}2}^{\text{ComBat}},\ldots ,e_{\mathrm{ijp}}^{\text{ComBat}}\right)}^{T}$ where p is the number of features. We then define these residuals using notation from Section 2.2 as

$$e_{\mathrm{ijv}}^{\text{ComBat}}=\frac{y_{\mathrm{ijv}}-{\hat{\alpha }}_{v}-x_{\mathrm{ij}}^{T}{\hat{\beta }}_{v}-\gamma_{\mathrm{iv}}^{*}}{\delta_{\mathrm{iv}}^{*}}$$

where i=1,2,…,M, $j=1,2,\ldots ,n_{i}$, M is the number of sites, and $n_{i}$ is the number of subjects acquired at site i. ${\hat{\alpha }}_{v}$, $x_{\mathrm{ij}}^{T}$, ${\hat{\beta }}_{v}$, $\gamma_{\mathrm{iv}}^{*}$, and $\delta_{\mathrm{iv}}^{*}$ are defined in Section 2.2.

*Step 2*. The $e_{\mathrm{ij}}^{\text{ComBat}}$ are assumed to have mean 0; their covariance matrices which we denote by $\sum_{i}$, however, may differ across sites. We first perform principal components analysis (PCA) on the full data residuals and represent the full data covariance matrix as $\sum =\sum_{k=1}^{q}\lambda_{k}\phi_{k}\phi_{k}^{T}$ where the rank $q=\min\left(\sum_{i=1}^{M}n_{i},p\right)$, $\lambda_{k}$ are the eigenvalues of ∑, and $\phi_{k}$ are the PCs obtained as the eigenvectors of ∑. In practice, PCA is performed on the sample covariance matrix $\hat{\sum }$ and we obtain estimated eigenvalues ${\hat{\lambda }}_{k}$ and eigenvectors ${\hat{\phi }}_{k}$. The ComBat‐adjusted residuals can then be expressed as $e_{\mathrm{ij}}^{\text{ComBat}}=\sum_{k=1}^{q}\xi_{\mathrm{ijk}}{\hat{\phi }}_{k}$ where $\xi_{\mathrm{ijk}}$ are the principal component scores.

We then aim to bring each within‐site covariance matrix $\sum_{i}$ to the pooled covariance across sites. Since our goal is to recover covariance structures resembling ∑, we approximate the within‐site covariance matrices as ${\hat{\sum }}_{i}=\sum_{k=1}^{q}{\hat{\lambda }}_{\mathrm{ik}}{\hat{\phi }}_{k}{\hat{\phi }}_{k}^{T}$ where ${\hat{\lambda }}_{\mathrm{ik}}$ are within‐site eigenvalues estimated as the sample variance of the principal component scores ${\hat{\lambda }}_{\mathrm{ik}}=\sum_{j=1}^{n_{i}}{\left(\xi_{\mathrm{ijk}}-\sum_{j=1}^{n_{i}}\xi_{\mathrm{ijk}}/n_{i}\right)}^{2}/\left(n_{i}-1\right)$ and ${\hat{\phi }}_{k}$ are estimated from the full data covariance. This model assumes that the covariance site effect is contained within the variances of the PC scores with the principal components estimated from the full data. This assumption may not hold in some cases, but harmonization of these PC score variances will bring the within‐site covariance matrices closer to the pooled covariance. This is analogous to how ComBat brings site‐specific variance closer to the variance estimated using observations across all sites.

*Step 3*. Thus, we posit:

$$\xi_{\mathrm{ijk}}=\mu_{\mathrm{ik}}+\rho_{\mathrm{ik}}\epsilon_{\mathrm{ijk}}$$

where $\epsilon_{\mathrm{ijk}}∼N\left(0,\tau_{k}^{2}\right)$, $\tau_{k}$ is the error standard deviation, and $\mu_{\mathrm{ik}}$, $\rho_{\mathrm{ik}}$ are the center and scale parameters corresponding to principal components k=1,2,…K where K≤q is a tuning parameter chosen to capture the desired proportion of the variation in the observations. If K is chosen such that K=q, all principal components are harmonized. Note that this is similar to the ComBat model, applied to each of the k principal component scores instead of the original measures. We can then estimate each of the K pairs of center and scale parameters by finding the values that bring each site's mean and variance in scores to the pooled mean and variance, which we denote ${\hat{\mu }}_{\mathrm{ik}})$ and ${\hat{\rho }}_{\mathrm{ik}}$. We then remove the site effect in the scores via $\xi_{\mathrm{ijk}}^{\text{CovBat}}=\left(\xi_{\mathrm{ijk}}-{\hat{\mu }}_{\mathrm{ik}}\right)/{\hat{\rho }}_{\mathrm{ik}}$.

*Step 4*. We obtain CovBat‐adjusted residuals $e_{\mathrm{ij}}^{\text{CovBat}}={\left(e_{\mathrm{ij}1}^{\text{CovBat}},e_{\mathrm{ij}2}^{\text{CovBat}},\ldots ,e_{\mathrm{ijp}}^{\text{CovBat}}\right)}^{T}$ by projecting the adjusted scores back into the residual space via,

$$e_{\mathrm{ij}}^{\text{CovBat}}=\sum_{k=1}^{K}\xi_{\mathrm{ijk}}^{\text{CovBat}}{\hat{\phi }}_{k}+\sum_{l=K+1}^{q}\xi_{\mathrm{ijl}}{\hat{\phi }}_{l}$$

We then add the intercepts and covariates effects estimated in Step 1 to obtain CovBat‐adjusted observations

$$y_{\mathrm{ij}v}^{\text{CovBat}}=e_{\mathrm{ijv}}^{\text{CovBat}}+{\hat{\alpha }}_{v}+x_{\mathrm{ij}}^{T}{\hat{\beta }}_{v}$$

### 2.4 Simulations

#### 2.4.1 Simulation settings

Let $y_{\mathrm{ij}}$, i=1,2,3, $j=1,2,\ldots ,n_{i}$ be vectors of length p representing simulated cortical thickness values for three sites, each with $n_{i}$ observations. The $y_{\mathrm{ij}}$ are generated using the following model:

$$y_{\mathrm{ijv}}=\alpha_{v}+x_{\mathrm{ij}}\beta_{v}+\gamma_{\mathrm{iv}}+\delta_{\mathrm{iv}}e_{\mathrm{ijv}}$$

where $x_{\mathrm{ij}}$ is a simulated diagnosis variable drawn from a Bernoulli distribution with probability 0.25, α is the first p/2 elements in each hemisphere from the sample mean vector of Scanner B observations in the ADNI data, β is the vector of simulated diagnosis effects on the mean, and $e_{\mathrm{ij}}$ is the vector of error terms. We simulate mean and variance site effects based on the assumptions of ComBat and CovBat. The mean site effects $\gamma_{i}={\left(\gamma_{i1},\gamma_{i2},\ldots ,\gamma_{\mathrm{ip}}\right)}^{T}$ are vectors drawn from independent and identically distributed (i.i.d.) normal distributions with mean zero and standard deviation 0.1. The variance site effects $\delta_{i}={\left(\delta_{i1},\delta_{i2},\ldots ,\delta_{\mathrm{ip}}\right)}^{T}$ are vectors drawn from site‐specific inverse gamma distributions with chosen parameters. For our simulations, we chose to distinguish the site‐specific scaling factors by assuming $\delta_{1v}\overset{i.i.d}{∼}\text{Inverse Gamma}\;\left(46,50\right)$, $\delta_{2v}\overset{i.i.d}{∼}\text{Inverse Gamma}\;\left(51,50\right)$, and $\delta_{3v}\overset{i.i.d}{∼}\text{Inverse Gamma}\;\left(56,50\right)$ for v=1,2,…,p.

### 2.5 Simple covariance effects

We first assess whether CovBat can recover the underlying covariance structure when the covariance site effects are captured by its PC directions. We refer to this simulation setting as the Simple Covariance Effects simulation. For p=62, we set the underlying covariance S as the sample correlation matrix of cortical thickness observations in the ADNI data, with eigendecomposition $S=\sum_{k=1}^{62}{\hat{\lambda }}_{k}{\hat{\phi }}_{k}{\hat{\phi }}_{k}^{T}$. We then generate error terms $e_{\mathrm{ij}}$ that contain site‐specific shifts in the first eigenvalue via

$$e_{\mathrm{ij}}∼N\left(0,S+c_{i}{\hat{\lambda }}_{1}{\hat{\phi }}_{1}{\hat{\phi }}_{1}^{T}\right)$$

where $c_{i}$ controls the severity of the covariance shift. For our simulations, we set $c_{1}=-1/2$, $c_{2}=0$, and $c_{3}=1/2$ so that the pooled covariance structure is equivalent to S. We choose $\beta_{i}=-0.5$ for $\left\lfloor p/4\right\rfloor$ regions of interest in both the left and right hemispheres to associate the simulated diagnosis with decreases in mean simulated cortical thicknesses.

We also investigate how the rank of the covariance effect influences CovBat harmonization results. We modify the rank of the simple covariance effect by varying K in the generation of error terms

$$e_{\mathrm{ij}}∼N\left(0,S+c_{i}\sum_{k=1}^{K}{\hat{\lambda }}_{k}{\hat{\phi }}_{k}{\hat{\phi }}_{k}^{T}\right)$$

where $c_{i}$ takes the same values as previously, $c_{1}=-1/2$, $c_{2}=0$, and $c_{3}=1/2$. We simulate datasets while choosing K as 2, 6, and 12 PCs and evaluate how harmonization influences detection of site via ML.

### 2.6 Complex covariance effects

To evaluate how CovBat performs when the covariance site effects are not easily captured by the principal components and when the simulated diagnosis may affect covariance, we modify the simulation to incorporate high‐rank covariance shifts due to site via $\Omega_{i}$ and due to simulated diagnosis as Ψ. For p=62, the error terms $e_{\mathrm{ij}}$ are now generated via

$$e_{\mathrm{ij}}∼N\left(0,D_{\mathrm{ij}}\sum_{\mathrm{ij}}D_{\mathrm{ij}}\right)$$

where $\sum_{\mathrm{ij}}=S+x_{\mathrm{ij}}\Psi +\Omega_{i}$, S is the sample correlation matrix of cortical thickness observations in the ADNI data, Ψ is a chosen diagnosis‐driven covariance shift matrix, and $\Omega_{i}$ are site‐specific covariance shift matrices. The matrices $D_{\mathrm{ij}}=\mathrm{diag}\left(d_{\mathrm{ij}}\right)$ where $d_{\mathrm{ijk}}=1/\sqrt{{\left(\sum_{\mathrm{ij}}\right)}_{\mathrm{kk}}}$ for k=1,2,…,62 ensure that these covariance effects do not modify the marginal variances of $e_{\mathrm{ij}}$. To constrain the covariance matrices to be positive definite, we set the negative eigenvalues of $\sum_{\mathrm{ij}}$ equal to a small constant, $10^{-12}$. For p<62, we instead generate $e_{\mathrm{ij}}$ from the p×p submatrices of $D_{\mathrm{ij}}\sum_{\mathrm{ij}}D_{\mathrm{ij}}$ constructed from the rows and columns corresponding to the first p/2 features in each hemisphere. In this simulation scenario, S is no longer the pooled covariance structure since the covariance site effects $\Omega_{i}$ can take any form and these site‐specific covariance structures do not necessarily combine across sites to resemble S. Instead of focusing on recovery of an underlying structure, we evaluate site effects throughout these simulations via ML.

We design four simulation experiments to test how CovBat performs in multiple settings with varied covariance effects. In our ComBat simulation, we generate data without any covariance site effect from a model that resembles the ComBat model. In the Diagnosis Affects Mean simulation, we then introduce a covariance site effect to assess if CovBat can outperform ComBat in harmonization of covariance. We next introduce a simulated diagnosis effect on covariance in the Diagnosis Affects Covariance simulation, which better illustrates how the detection of the simulated diagnosis is affected by related covariance effects. Finally, we design a Covariance Only simulation, which has no site or diagnosis effects in mean or variance, but still contains site and diagnosis effects in covariance. This final simulation illustrates how effects on covariance can influence ML results and be addressed through our proposed harmonization method.

In the ComBat simulation, we impose site effects in mean and variance while having the simulated diagnosis affect only the mean of the observations. We choose $\beta_{i}=-0.5$ for $\left\lfloor p/4\right\rfloor$ regions of interest in both the left and right hemispheres to impose that about half of the ROIs are negatively associated with the simulated diagnosis. We also choose the $\Omega_{i}$ and Ψ to be 62×62 zero matrices to ensure that the covariance does not depend on site or the simulated diagnosis.

In the Diagnosis Affects Mean simulation, we again impose that the simulated diagnosis only affects the mean of the measurements but also introduce a site effect in covariance. We keep the same β and Ψ as in the ComBat simulation. However, we choose $\Omega_{i}$ to be distinct 62×62 high‐rank matrices to distinguish covariance structures across sites. These $\Omega_{i}$ are constructed by downsampling three distinct images to 62 × 62 pixels and then scaling the values so that the diagonal is a vector of ones. These matrices thus have different eigenvectors from each other and also from the sample covariance matrix S, which simulates complex site covariance structures which do not have the same eigenvectors.

In the Diagnosis Affects Covariance simulation, we assume that the simulated diagnosis affects not only mean, but also covariance. We choose the diagnosis effect on covariance to be proportional to a site's covariance shift. This scenario represents a situation where detection of the diagnosis using ML could be highly influenced by the presence of site effects. We use the same β value as in the ComBat and diagnosis affects mean simulations but choose Ψ to be related to $\Omega_{3}$ to force confounding of Site 3 and diagnosis effects on covariance. To achieve this, we set $\Psi =-\left(3/4\right)\Omega_{3}$.

In the Covariance Only simulation, we assume that both site and the diagnosis influence the covariance, not the mean or variance. We fix γ=0 and δ=1 to remove site effects in mean and variance while also using the same $\Omega_{i}$ as in the Diagnosis Affects Mean and Diagnosis Affects Covariance simulations. Furthermore, we modify the diagnosis effect by setting β=0 while keeping $\Psi =-\left(3/4\right)\Omega_{3}$ for the diagnosis effect in covariance.

### 2.7 Simulation experimental design

In our simple covariance effects simulation with rank one covariance effects, we generate 1,000 datasets across varying sample sizes and number of features to evaluate recovery of the underlying covariance structure. For each site, we calculate the average Frobenius distance across datasets between each sample within‐site covariance matrix and the true covariance S. We then report the average across sites before and after harmonization, where CovBat harmonization is performed on PCs that explain 95% of the variation.

For the other simulation settings, we generate 1,000 datasets for several choices of within‐site sample size and number of features and perform experiments to evaluate detection of site and disease using ML. For each dataset, we (a) randomly split the sample into 50% training data and 50% validation data, (b) train a random forests algorithm to recognize if the observations either are from Site 1 or have the simulated diagnosis, and (c) assess predictive performance on the testing data. Our random forests algorithm is implemented via the *randomForest* package on CRAN with default parameters using R version 3.6.1. Lower AUC for detection of Site 1 and higher AUC for detection of the simulated diagnosis indicate improved harmonization. CovBat harmonization is performed on PCs that explain 95% of the variation. For prediction of Site 1, we avoid the possibility that site could be detected through the simulated diagnosis by using linear models to residualize out disease from each simulated cortical thickness value. For simulated datasets where the training set does not contain observations with the simulated diagnosis, the random forest algorithm cannot be trained so we generate another dataset to replace it. We repeat these ML experiments while varying the number of PCs included in the CovBat harmonization. For CovBat including PCs that explain 95% of the variation, we also perform MANOVA for testing associations with site and simulated diagnosis and report the rejection rate at a type I error rate of 0.05 across the 1,000 datasets.

### 2.8 Recovery of covariance

**FIGURE 1:**
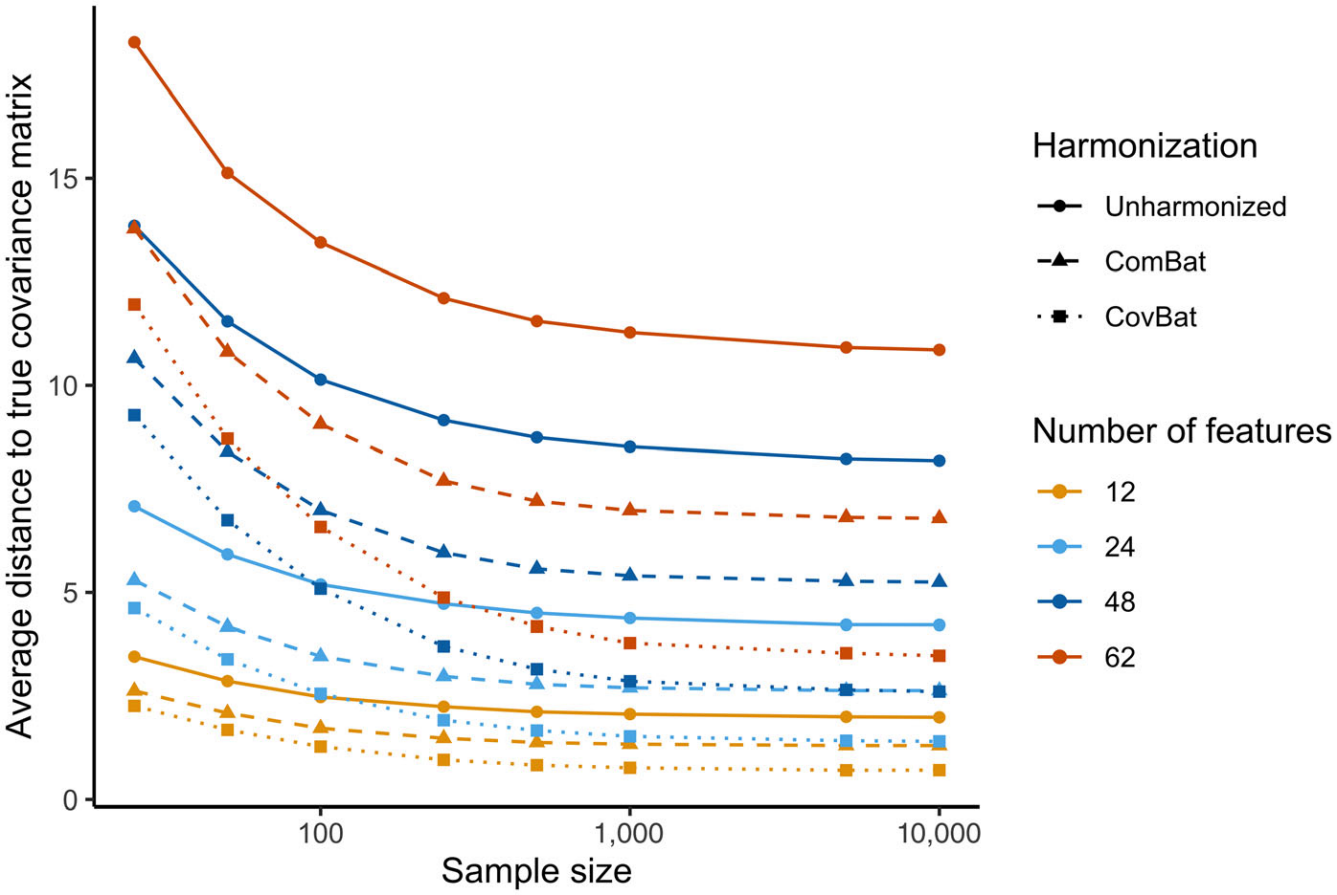
Average across sites of the Frobenius distance between sample site‐specific covariance matrices and the true covariance matrix for the Simple Covariance Effects simulation. The displayed values are averaged across the mean Frobenius distance for each site, which are taken across 1,000 simulations each. Results are plotted for a sample size per site of 25, 50, 100, 250, 500, 1,000, 5,000, and 10,000

We first perform simulations to assess whether either harmonization method can recover the underlying covariance structure in our simple simulation setting and harmonize covariance matrices generally. For ComBat and CovBat, we include our simulated diagnosis status as a covariate. We apply our CovBat method using the number of PCs that explain 95% of the variation. In the Simple Covariance Effects simulation, Figure 1 shows that CovBat outperforms ComBat in recovery of the true covariance structure (denoted S in Section 2.5) across all parameters considered. Remaining deviation from the true covariance can be explained by error in covariance estimation; even with 10,000 samples per site and 62 features the distance of the pooled covariance estimate from the true covariance is still 3.14. In Table 1, we observe in the same simulation setting with a within‐site sample size of 250 and 62 simulated features that CovBat performs best in harmonizing within‐site covariance matrices. That result is replicated with a more complex covariance site effect in the Diagnosis Affects Mean simulation as shown in Table 2.

**TABLE 1:** Mean and standard deviation of pairwise Frobenius norms between within‐site covariance estimates for the simple covariance effects simulation

	Unharmonized	ComBat	CovBat
1,2	16.85 (3.5)	11.21 (2)	4.27 (0.5)
1,3	30.08 (4.9)	16.78 (2.2)	4.62 (0.6)
2,3	15.65 (4.7)	7.17 (1.7)	4.17 (0.5)

*Note*: Standard deviations are reported in parentheses.

**TABLE 2:** Mean and standard deviation of pairwise Frobenius norms between within‐site covariance estimates for the diagnosis affects mean simulation

	Unharmonized	ComBat	CovBat
1,2	15.77 (2.1)	15.39 (1.4)	12.81 (0.8)
1,3	13.22 (1.4)	13.68 (1.3)	11.69 (0.7)
2,3	12.79 (1.8)	10.98 (0.7)	10.98 (0.5)

*Note*: Standard deviations are reported in parentheses.

### 2.9 Detection of site and diagnosis

**FIGURE 2:**
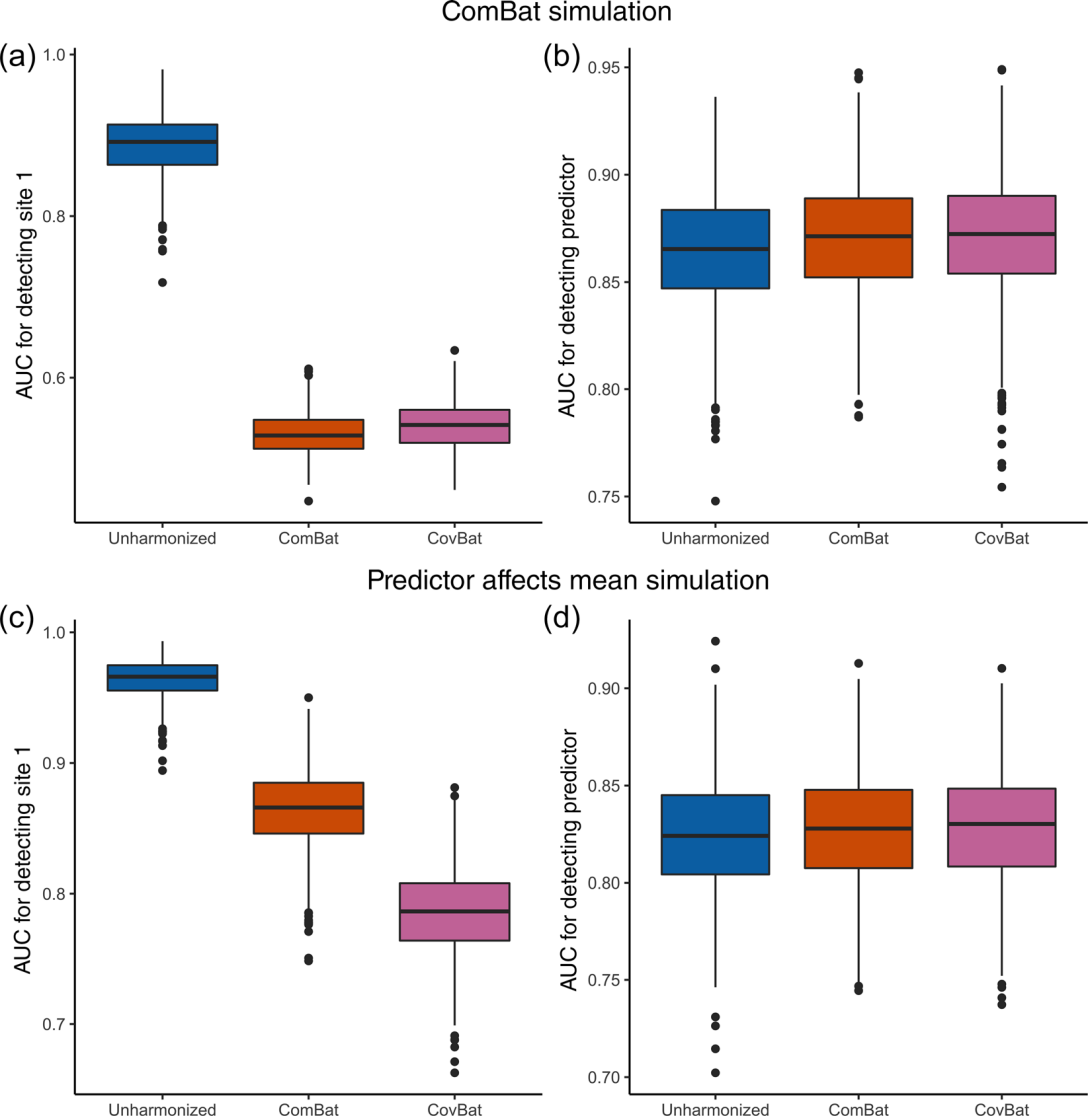
Results from ML simulations for detection of site and for detection of the simulated diagnosis in the absence of simulated diagnosis effects on covariance. The simulated data consists of 62 cortical thicknesses for 250 subjects per site across three sites. For each of 1,000 simulations, the data is randomly split into 50% training and 50% validation. A random forests algorithm is trained using the training set to predict either Site 1 or the presence of the simulated diagnosis. (a), Boxplot showing Site 1 detection in the ComBat simulation. (b), Boxplot showing simulated diagnosis detection in the ComBat simulation. (c), Boxplot showing Site 1 detection in the diagnosis affects mean simulation. (d), Boxplot showing simulated diagnosis detection in the diagnosis affects mean simulation

We then evaluate CovBat through two ML experiments across all simulation settings considered. For our main analyses, we simulate 62 cortical thicknesses for 250 subjects per site. We begin by examining a ComBat simulation, where we generate data from the original ComBat model. That is, we impose mean and variance site effects while also simulating a diagnosis that has an effect on the mean. Figure 2a,b show that CovBat performs almost identically to ComBat in this scenario, showing that our method performs competitively in the absence of covariance effects. In the Diagnosis Affects Mean simulation, Figure 2c,d shows that CovBat also substantially reduces the chance of detecting site and performs similarly to ComBat for detection of the simulated diagnosis. These simulations demonstrate that CovBat performs at least as well or better than ComBat when the simulated diagnosis affects only the mean of the observations.

**FIGURE 3:**
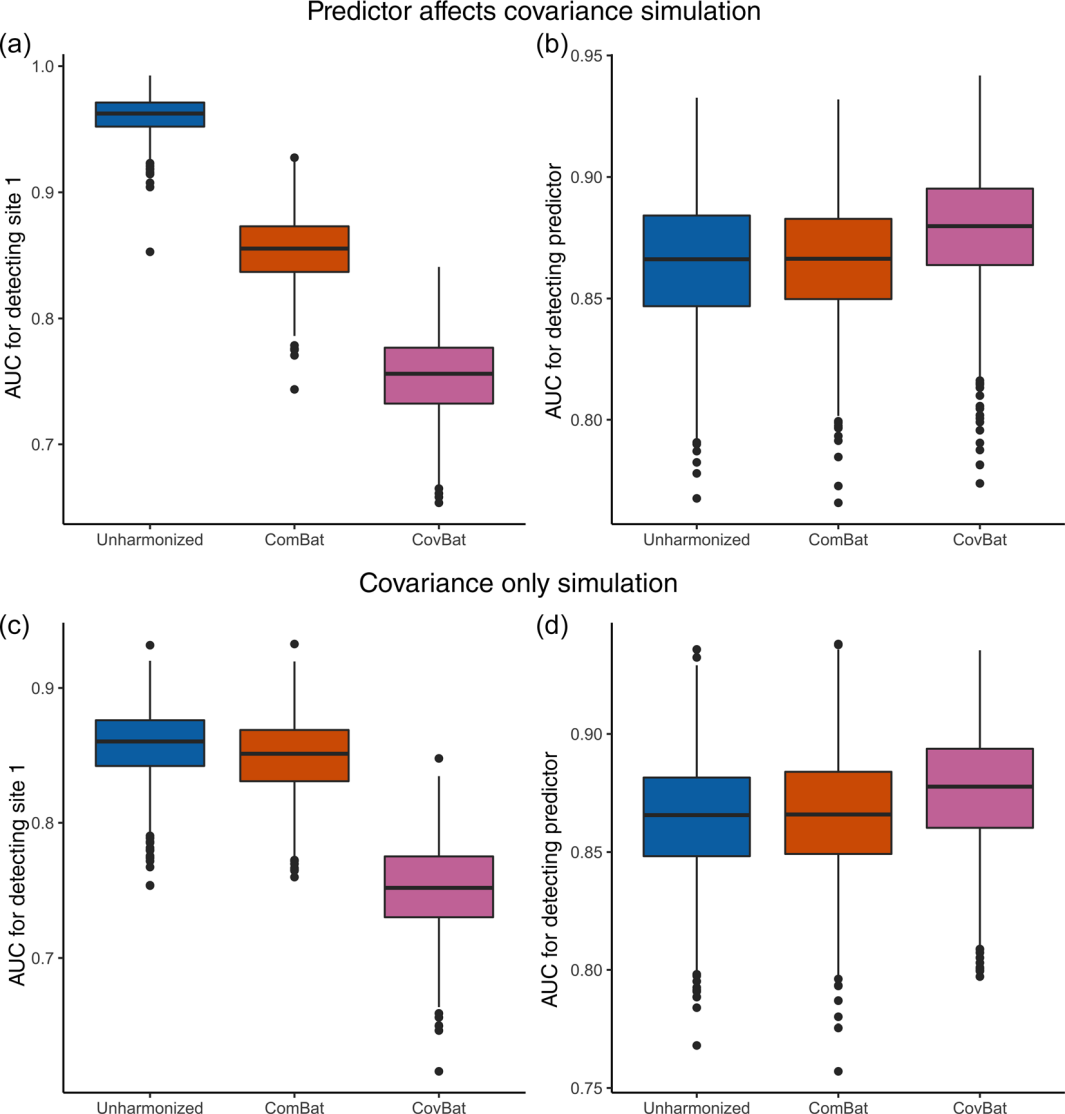
Results from ML simulations for detection of site and for detection of the simulated diagnosis where the simulated diagnosis also confounds covariance. The simulated data consists of 62 cortical thicknesses for 250 subjects per site across three sites. For each of 1,000 simulations, the data is randomly split into 50% training and 50% validation. A random forests algorithm is trained using the training set to predict either Site 1 or the presence of the simulated diagnosis. (a), Boxplot showing Site 1 detection in the Diagnosis Affects Covariance simulation. (b), Boxplot showing simulated diagnosis detection in the diagnosis affects covariance simulation. (c), Boxplot showing Site 1 detection in the covariance only simulation. (d), Boxplot showing simulated diagnosis detection in the Covariance Only simulation

We then incorporate a diagnosis effect on covariance in our Diagnosis Affects Covariance simulation and show that CovBat reduces detection of site (Figure 3a) and maintains the association with the simulated diagnosis (Figure 3b). In order to further emphasize the importance of covariance effects, we investigate a Covariance Only simulation where both site and diagnosis effects exist only in the covariance of observations, but not the mean or variance. In unharmonized data, we observe high mean AUC values for detection of Site 1 and detection of the simulated diagnosis, both of which are essentially unaffected after implementing ComBat. After CovBat though, we see substantial improvements on both metrics (Figure 3c,d). While CovBat performs well in both simulations, we find that CovBat does not entirely remove the severe covariance site effect. Regardless, we observe that CovBat offers notable improvements over ComBat owing to its harmonization of covariance.

### 2.10 Performance across sample properties

We then conduct additional analyses to assess the robustness of CovBat to reductions in sample size per site and number of features. In Table S1, we show that the random forests largely hold for simulations without a diagnosis effect on covariance; however, in the absence of a covariance site effect CovBat performs slightly worse overall and in situations with small sample size per site ($n_{i}=25$) and larger number of features (p≥48) we observe that CovBat can inflate detection of site. Table S2 shows that similar results hold in simulations with a diagnosis effect on covariance with CovBat showing good performance overall but poor performance with small sample sizes and large number of features. We also show in Table S3 through the Covariance Only simulation that site and diagnosis can both be detected even without affecting the mean of observations, as demonstrated by high AUCs for detection across all settings. To assess if our findings may be tied to the ML paradigm, we additionally perform MANOVA for site and diagnosis status across Diagnosis Affects Mean and Diagnosis Affects Covariance simulations and show in Tables S4 and S5 that associations with site are reduced after CovBat across all scenarios while associations with diagnosis are preserved. We repeat these MANOVA analyses in the Simple Covariance Effects simulation and show in Table S6 that CovBat performs better than ComBat at controlling the rejection rate across nearly all parameters considered. However, CovBat only successfully controls the rejection rate at 5% in simulations with less features, small sample size, and large sample size. The controlled rejection rate in small samples however could be explained by the low power of MANOVA in small sample size relative to the number of features (Stevens, 1980).

### 2.11 Choosing the number of PCs in CovBat

To better inform the choice of PCs in CovBat, we evaluate the simulation results obtained across varying number of PCs. For simple covariance effects with varying low rank structures, Figure S2 shows that harmonizing smaller numbers of PCs yields suboptimal results for reducing the chance of detecting sites. Across varying ranks of simulated site effects contained in the covariance structures (2–12 PCs), simulation results show that the median AUC for detecting site achieves the lowest value when CovBat includes PCs that explain around 90% of total variation for a sample size of 100. With a larger sample size of 250, the best performance was achieved when CovBat includes PCs that explain 95% of the total variation. Across both sample sizes, the U‐shape curves in median AUC for detecting sites indicate that including excess numbers of PCs in CovBat hurts the performance due to overfitting and the optimal number of PCs increases with sample size. For the high rank covariance effect in our Diagnosis Affects Covariance, we observe in Figure S3 that for a sample size of 250 increasing the number of PCs lowers the AUC for detection of site across the whole range of considered PCs. However, looking at a sample size of 50, we observe that AUC for site detection increases as we select PCs that explain between roughly 95% and 100% of the variation. For detection of simulated diagnosis, Figure S4 shows that CovBat largely maintains detection of diagnosis across all numbers of PCs included and sample sizes, with minor increases in median AUC as more PCs are included.

## 3 ADNI DATA ANALYSIS

### 3.1 Harmonization

We evaluate the performance of harmonization in the ADNI data by comparing three different approaches. First, we test the original unharmonized data in our subsequent experiments. Second, we produce ComBat‐harmonized data by performing ComBat while including age, sex, and diagnosis status as covariates. Third, we obtain CovBat‐harmonized data by running CovBat with varying number of PCs, while also including age, sex, and diagnosis status. For our primary analyses, we run CovBat while including 37 PCs, which cumulatively explain 95% of variation. We include additional analyses evaluating how our results vary with the choice of PCs.

### 3.2 ML experiments

Using the full ADNI sample, we evaluate whether the harmonization procedures affect the results of ML using the neuroimaging measures as patterns for a prediction algorithm. We achieve this through a Monte Carlo split‐sample experiment where we (i) randomly split the subjects into 50% training set and 50% validation set, (ii) train a random forests algorithm to detect either scanner manufacturer or a binary clinical covariate, and (iii) assess predictive performance on the validation set via AUC. Our random forests algorithm is implemented via the *randomForest* package on CRAN with default parameters using R version 3.6.1. We train separate models for unharmonized, ComBat‐harmonized, and CovBat‐harmonized data where both harmonization methods are performed including age, sex, and diagnosis status as covariates. We perform steps (i)–(iii) 100 times for each dataset and report the mean AUC along with standard deviation. For these experiments, lower AUC for detection of scanner manufacturer and higher AUC for detection of clinical covariates would indicate improved harmonization. For prediction of scanner manufacturer, we avoid the possibility that scanner could be detected through the covariates age, sex, and disease status by residualizing out these variables from each cortical thickness value via linear models. To assess performance across choices of PCs in CovBat, we repeat our ML experiments across varying numbers of PCs explaining between 44 and 100% of the variation.

### 3.3 Classical multivariate analyses

We also evaluate the harmonization methods using multivariate analysis of variance (MANOVA). MANOVA tests for differences in mean across groups in multivariate data, but is known to be sensitive to differences in covariance across groups. We perform MANOVA across scanner manufacturer, sex, and diagnosis status using Pillai's trace, which is known to be more robust to inhomogeneity in covariance than other alternative test statistics (Olson, 1974). We report p‐values for these associations before and after harmonization.

### 3.4 CovBat reduces covariance site effects

**FIGURE 4:**
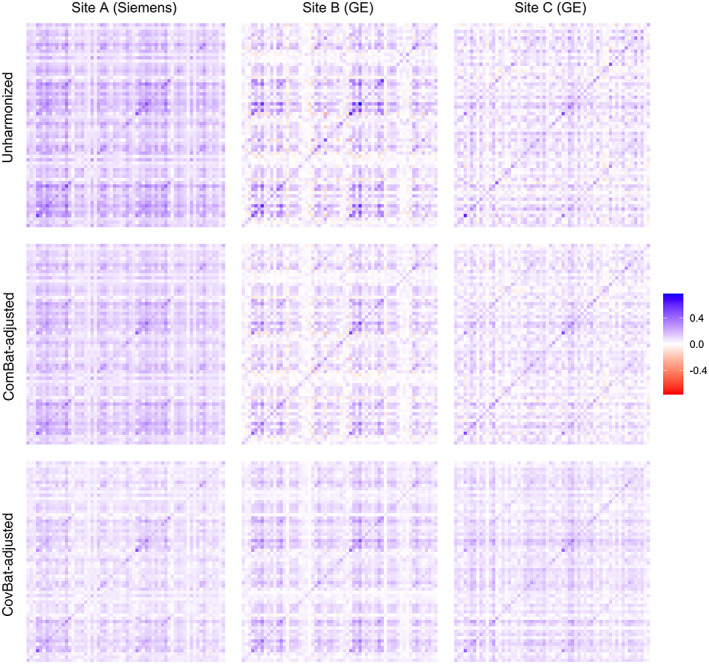
Covariance matrices for cortical thickness values acquired on three sites before and after harmonization. All covariance matrices are estimated after residualizing the data on age, sex, and diagnosis status. Site A uses a Siemens Symphony 1.5T scanner with 23 subjects and the other sites use General Electric Signa Excite 1.5T scanners with 20 subjects each

We first examine the empirical covariance of the ADNI data before and after harmonization. To evaluate site differences, we investigate the three largest ADNI sites. Site A consists of 23 subjects acquired on a Siemens Symphony 1.5T scanner while Sites B and C each consist of 20 subjects acquired on GE Signa Excite 1.5T scanners. See Table 3 for demographic details. To avoid influence of site demographics on the covariance matrices, we residualize the cortical thickness measures across the three sites jointly on age, sex, and diagnosis status in each dataset using a linear model. Figure 4 shows the covariance matrices for each site using the residualized cortical measures both before and after harmonization (ROI labels are shown in Figure S1). The differences between the unharmonized covariance matrices are striking. Especially notable are the increased positive covariance across most pairs of cortical regions in Site A and the weakened correlation between the right and left hemispheres in Site C, visible as the diagonal line in the top‐left and bottom‐right quadrants. Visually, the covariance differences remain similar after applying ComBat. These inter‐site differences are considerably mitigated after CovBat.

**TABLE 3:** ADNI demographics by site for the three sites with the largest number of acquired subjects

	A (Siemens)	B (General Electric)	C (General Electric)	*p*
Number of subjects	23	20	20	
Age (mean [*SD*])	74.48 (5.13)	76.90 (8.18)	78.78 (6.19)	.11
Diagnosis (%)				.57
AD	7 (30.4)	6 (30.0)	5 (25.0)	
CN	6 (26.1)	5 (25.0)	2 (10.0)	
LMCI	10 (43.5)	9 (45.0)	13 (65.0)	
Male (%)	10 (43.5)	13 (65.0)	16 (80.0)	.05

*Note*: Manufacturer of each site's scanner is displayed in parentheses. ANOVA p‐values for testing differences in the mean of continuous variables and Chi‐squared test p‐values for testing the differences in categorical variables are reported in the rightmost column.

Abbreviations: AD, Alzheimer's disease; CN, cognitively normal; LMCI, late mild cognitive impairment.

We also provide quantitative comparisons for pairwise distances across sites before and after harmonization in Table 4. A tuning parameter of the CovBat model is the desired proportion of variance explained in the dimension reduction space, which we select at 95% (37 PCs). To ensure that our results do not depend strongly on the choice of tuning parameter, we also report the minimum and maximum of the pairwise Frobenius norms after applying CovBat with percent variation explained ranging from 44% (2 PCs) to 100% (62 PCs). We report the results of this sensitivity analysis in parentheses. We find that ComBat adjustment can modestly harmonize the covariance matrices but CovBat adjustment shows large reductions in the between‐site distances across a range of tuning parameter choices.

**TABLE 4:** Pairwise distances between site‐specific covariance matrices

	Unharmonized	ComBat	CovBat
A,B	5.39	4.29	2.60 (2.59–2.90)
A,C	5.82	4.27	2.50 (2.49–2.76)
B,C	4.69	3.32	2.67 (2.65–3.18)

*Note*: Differences in covariance structure between sites are reported as the Frobenius distance between covariance matrices calculated across observations acquired on each site. Results from adjusting the number of PC scores ranging from those explaining 44–100% of variation are shown in parentheses as the minimum and maximum pairwise Frobenius norms across the range.

### 3.5 CovBat impairs detection of site

**FIGURE 5:**
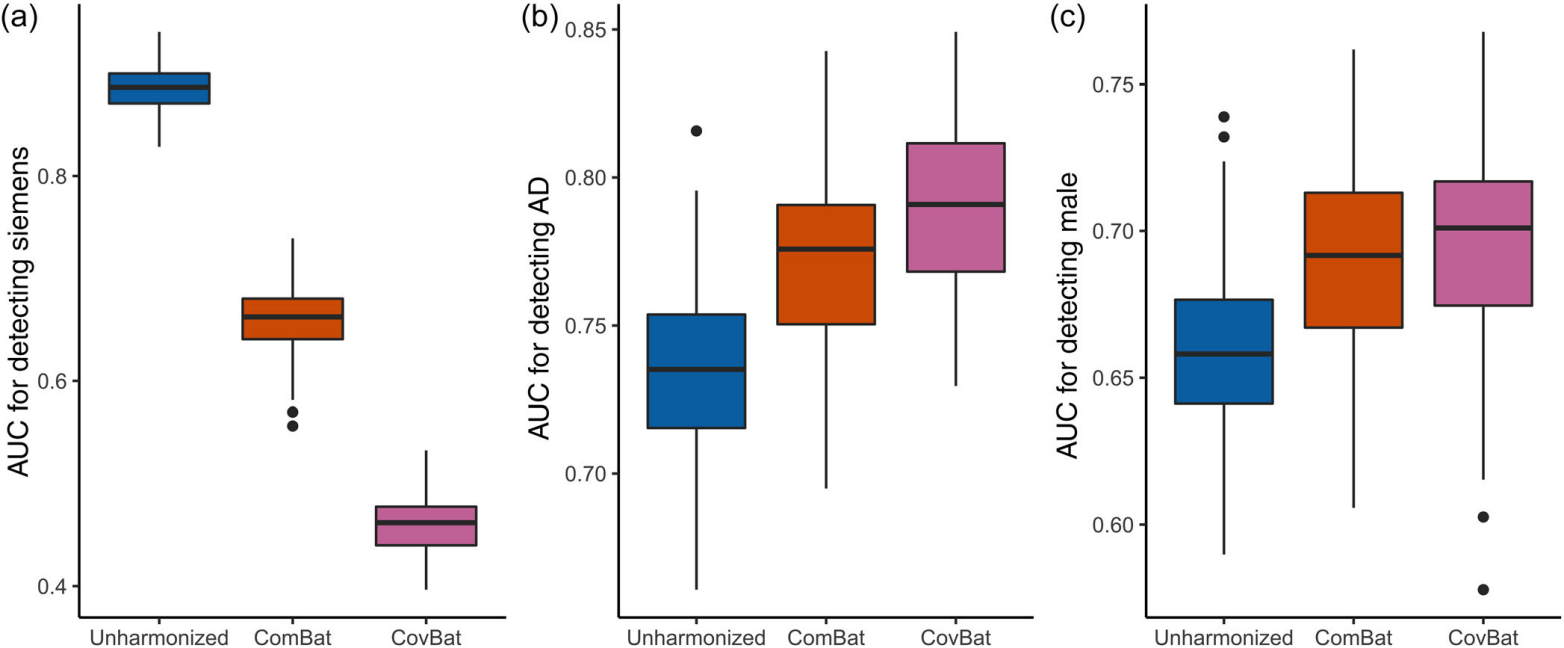
Multivariate pattern analysis experiments for detection of scanner manufacturer, sex, and Alzheimer's disease status using cortical thickness data. The data are randomly split into 50% training and 50% validation then used to train a random forests algorithm to predict a specified trait. AUC values from 100 repetitions of this analysis are reported for unharmonized, ComBat‐adjusted, and CovBat‐adjusted data. (a) Boxplot showing results for detecting if subjects were acquired on a Siemens scanner. Results for detection of Alzheimer's disease status are shown in (b) and results for detection of sex are shown in (c)

To evaluate the potential impact of site effects in covariance using ML, we conduct a Monte‐Carlo split‐sample experiment for prediction of scanner manufacturer labels using all 213 ADNI subjects before and after harmonization with existing methods. We train using data harmonized with the state‐of‐the‐art ComBat method and our proposed method, CovBat. Figure 5a shows that Siemens sites are easily identifiable based on unharmonized cortical thickness measurements (median area‐under‐the‐curve [AUC] 0.89, IQR 0.87–0.90), which is consistent with recent findings (Glocker et al., 2019). We also note that scanner manufacturer is still detected after ComBat is applied (0.66, 0.64–0.68). After CovBat, the ML method's performance for differentiating between sites is close to chance (0.46, 0.44–0.48). CovBat's performance depends on the number of PCs included in the model, but Figure S5 shows that the performance gain for each PC becomes negligible around the number of PCs that explain 95% of the variation. DeLong's test results shown in Figure S2 suggest that these AUC values for site detection are significantly different between ComBat and CovBat. Using MANOVA, Table 5 shows that the association with scanner manufacturer is statistically significant in unharmonized and ComBat‐adjusted data but is eliminated in CovBat‐adjusted data.

**TABLE 5:** Multivariate analysis of variance p‐values for scanner manufacturer, sex, and Alzheimer's disease status using cortical thickness data

	Unharmonized	ComBat	CovBat
Manufacturer	<.001	<.001	1
Sex	<.001	<.001	<.001
Diagnosis	<.001	<.001	<.001

*Note*: The analysis is performed using Pillai's trace as the test statistic.

### 3.6 CovBat retains biological associations

It is well‐known that cortical thickness differs substantially by sex and AD status (Lerch et al., 2005; Sowell et al., 2007). To assess whether CovBat maintains biological associations of interest, we perform two ML experiments using the full ADNI data to classify healthy versus AD and to differentiate patients by sex. Figure 5b,c show that both of these biological associations are retained after either harmonization method. For AD classification, the median AUC increases from 0.74 (IQR 0.72–0.75) in unharmonized data to 0.78 (0.75–0.79) in ComBat‐adjusted data to 0.79 (0.77–0.81) in CovBat‐adjusted. Similarly, the median AUC for detection of sex increased from 0.66 (0.64–0.68) to 0.69 (0.67–0.71) to 0.70 (0.67–0.72). For detection of both AD and male sex, DeLong's test results plotted in Figure S6 support that the AUCs are not significantly different between ComBat and CovBat. These findings suggest that CovBat not only provides thorough removal of site effects, but also maintains clinical associations. Figure S4 shows that CovBat retains these associations across varying number of PCs included in the model. Appendix A1 shows that similar results hold for prediction of age, where both ComBat and CovBat reduce root‐mean‐square error for prediction of age compared to the unharmonized data. Appendix A2 shows that these results for both detection of site and biological associations largely hold even when CovBat is trained on a subset of the data and all sites are included in both the training and validation sets. MANOVA results in Table 5 show that the significant associations with diagnosis and sex are retained after either harmonization method.

## 4 DISCUSSION

The growing number of multi‐site studies across diverse fields has spurred the development of harmonization methods that are general, but also account for field‐specific challenges. In neuroimaging research, the rise of ML in neuroimaging has established an unmet need for harmonization of covariance. We demonstrate that strong site effects in covariance exist, influence downstream ML experiments, and remain after performing the state‐of‐the‐art harmonization. We then propose a novel method and demonstrate that it is effective in removing site differences in covariance and retaining the detection of biological associations via ML. Simulation studies show similar ML results and demonstrate that CovBat performs well across a variety of settings and sample sizes.

In ADNI data, we show that substantial differences exist in the covariance structures of cortical thickness observations and can be mitigated through our proposed method. We furthermore show that ML can detect these site effects, whether through ML or conventional multivariate analyses. These results mirror recent studies that predict scanner from neuroimaging features with high accuracy (Glocker et al., 2019) and a recent study demonstrating that ComBat is insufficient to prevent detection of Siemens‐manufactured scanners in a large multi‐site dataset (Nielson et al., 2018). We then demonstrate that CovBat can almost entirely prevent site detection in the ADNI dataset. To ensure generalizability of these results, implementation of CovBat in other multi‐site studies of varying experimental designs should be pursued in the future.

In simulation, CovBat shows generally strong performance in removing site effects in medium and large sample sizes across varying number of features and complexity of the site effect. We demonstrate that CovBat almost fully removes covariance site effects when they exist in the principal component directions, but deviations from the true covariance still remain due to error in covariance estimation, overcorrection of PCs without site difference, and the remaining site effects in the marginal variances. Additionally, ML results show that considerable site effects may remain in more complex scenarios. Caution should be taken in attempting to address covariance site effects in smaller samples with many features. We show potential increases in site detection in these situations, which are potentially the result of poor covariance estimation in high‐dimensional settings. Through investigating the performance of CovBat across varying number of PCs being harmonized, we conclude that the chance of detecting sites could increase when including excessive number of PCs in cases with simple site covariance effects or small sample size relative to the number of features. We generally recommend selecting number of PCs that cumulatively explain 90–95% of the total variation depending on the sample size. While we do not observe these limitations through MANOVA, we also acknowledge that MANOVA may be underpowered in our scenarios with low sample size and high dimensionality as shown in previous studies (Stevens, 1980).

Our proposed method harmonizes covariance across sites by removing mean and variance shifts in the principal components space, which we show to be effective in addressing the covariance effects we observe. This idea resembles spectral models, which also relate covariates to the eigendecomposition of covariance matrices (Boik, 2002). Our method assumes that the ideal covariance structure exists in the eigenspace of the full data covariance matrix. As we show through our simulations, in some cases this model may be insufficient to remove site effects, which do not resemble the covariance structure of the full data. Potential extensions could incorporate methods that model site effects as separate low‐rank structures (Hoff & Niu, 2012) or identify projections most related to site (Zhao, Wang, Mostofsky, Caffo, & Luo, 2019). However, implementation of these methods in a harmonization framework may not be as straightforward as our proposed method.

A limitation of our methodology is that CovBat is a covariate‐assisted harmonization method similar to ComBat and requires specification of covariates to protect in the data. Associations with covariates not included in the harmonization step can certainly be removed alongside site effects. Furthermore, nonlinear covariate associations may not be adequately captured by the linear model and future extensions of CovBat could consider incorporating previous work on general additive models in ComBat to capture complex covariate effects (Pomponio et al., 2020). Recent articles have identified situations where spurious associations can be introduced via ComBat (Nygaard, Rødland, & Hovig, 2016; Zindler, Frieling, Neyazi, Bleich, & Friedel, 2020). While we do not observe CovBat introducing false positives in our investigation, care must be taken in implementing CovBat protecting for the outcome of interest especially in unbalanced study designs. We reiterate previous advice that analyses should be performed with and without harmonization and the analysis design be made very clear to ensure that results can be interpreted properly (Zindler et al., 2020).

Our study demonstrates that site effects can exist in the covariance of structural neuroimaging data and can be mitigated via our proposed methodology. Future studies should determine how scanner properties can influence the covariance structure of the data and if other multi‐site multivariate neuroimaging studies contain similar effects. Further methodological work could utilize other covariance modeling strategies in order to address more complex site effects. Since our method operates on general multivariate data, our findings extend directly to functional, metabolic, and other imaging modalities. However, our method does not currently handle time series from functional imaging, which could be the subject of future investigation. Further studies should also determine the extent to which multivariate statistical and ML studies of genomic data are susceptible to the biases documented.

### 4.1 Software

All of the postprocessing analysis was performed in the R statistical software (V3.6.1). CovBat is available for both R and Python (https://github.com/andy1764/CovBat_Harmonization). Reference implementations for ComBat are available in R and Matlab (https://github.com/Jfortin1/ComBatHarmonization) and in Python (https://github.com/ncullen93/neuroCombat). Our R implementation of CovBat runs in reasonable time, even in large samples. We generate observations with 62 features with varying sample sizes. On a MacBook Pro (16‐in., 2019) with a 2.3 GHz 8‐Core Intel Core i9 and 32 GB 2667 MHz DDR4 memory, CovBat runs in 0.014 s for 100 samples, 0.090 s for 1,000 samples, 1.088 s for 10,000 samples, and 12.235 seconds for 100,000 samples.

## CONFLICT OF INTEREST

The authors declare no potential conflict of interest.

## AUTHOR CONTRIBUTIONS

Russell T. Shinohara and Haochang Shou contributed equally to this work. **Andrew A. Chen:** formal analysis, conceptualization, methodology, software, investigation, writing—original draft. **Joanne C. Beer:** data curation, writing—review & editing. Nicholas J. Tustison: data curation, Writing—review & editing. **Philip A. Cook:** data curation, writing—review & editing. **Russell T. Shinohara:** supervision, conceptualization, methodology, writing—review & editing. **Haochang Shou:** supervision, conceptualization, methodology, writing—review & editing.

## Supporting information


**Figure S1** Covariance matrix for cortical thickness values acquired at the largest site. Site A is a Siemens Symphony 1.5T scanner. Regions of interest are labeled on the *x*‐axis and *y*‐axis.
**Figure S2**: Simple Covariance Effect simulation AUCs for detection of site after applying CovBat including varying numbers of principal components. Each point represents the median and interquartile range of AUCs for detection of site across 1,000 simulations. The rank of the site covariance effect varies in each plot and the number of features is fixed at 62. Results are displayed for both 100 and 250 samples as separate lines.
**Figure S3**: Diagnosis Affects Covariance simulation AUCs for detection of site after applying CovBat including varying numbers of principal components. Each point represents the median and interquartile range of AUCs for detection of site across 1,000 simulations. Differing sample sizes and numbers of features are represented by different lines in each plots.
**Figure S4**: Diagnosis Affects Covariance simulation AUCs for detection of simulated diagnosis after applying CovBat including varying numbers of principal components. Each point represents the median and interquartile range of AUCs for detection of diagnosis across 1,000 simulations. Differing sample sizes and numbers of features are represented by different lines in each plots.
**Figure S5**: AUC for machine learning detection after CovBat across varying numbers of PCs included in the model. For each number of PCs, the median and IQR for detection of either Siemens, male, or Alzheimer's disease are displayed. Each point represents 1,000 splits of the ADNI data into 50% training and 50% validation.
**Figure S6**: DeLong's test *p*‐value *Q*–*Q* plots for comparing AUC between ComBat and CovBat. For each of the 100 train‐test splits within each MVPA experiment, DeLong's test is performed with the two‐sided null hypothesis that the AUC is different in ComBat‐adjusted and CovBat‐adjusted data. The p‐values for each experiment are compared to a uniform distribution from 0 to 1.
**Table S1**: AUC results from MVPA simulations for detection of site across multiple sample sizes (*n*) and number of features (*p*) in the absence of predictor effects on covariance. For each of 1,000 simulations, the data is randomly split into 50% training and 50% validation then a random forests algorithm is trained using the training set to predict either Site 1 or the presence of the binary predictor. The median AUC across these simulations are reported with lower and upper quartiles displayed in parentheses. Scenarios where CovBat outperforms ComBat are colored in blue.
**Table S2**: AUC results from MVPA simulations for detection of site across multiple sample sizes (*n*) and number of features (*p*) where the predictor has effects on covariance. For each of 1,000 simulations, the data is randomly split into 50% training and 50% validation then a random forests algorithm is trained using the training set to predict either Site 1 or the presence of the binary predictor. The median AUC across these simulations are reported with lower and upper quartiles displayed in parentheses. Scenarios where CovBat outperforms ComBat are colored in blue.
**Table S3**: AUC results from MVPA simulations for detection of simulated diagnosis across multiple sample sizes (*n*) and number of features (*p*) where the predictor has effects on covariance. For each of 1,000 simulations, the data is randomly split into 50% training and 50% validation then a random forests algorithm is trained using the training set to predict either Site 1 or the presence of the binary predictor. The median AUC across these simulations are reported with lower and upper quartiles displayed in parentheses.
**Table S4**: MANOVA rejection rates for associations with site and diagnosis status across multiple sample sizes (*n*) and number of features (*p*) in the Diagnosis affects Mean simulations. For each of 1,000 simulations, MANOVA is performed separately for site and diagnosis using Pillai's trace. The rejection rate across these simulations is reported as the percentage of *p*‐values less than .05.
**Table S5**: MANOVA rejection rates for associations with site and diagnosis status across multiple sample sizes (*n*) and number of features (*p*) in the Diagnosis affects Covariance simulations. For each of 1,000 simulations, MANOVA is performed separately for site and diagnosis using Pillai's trace. The rejection rate across these simulations is reported as the percentage of *p*‐values less than .05. Scenarios where the rejection rate is less than 0.05 are colored in blue.
**Table S6**: MANOVA rejection rates for associations with site and diagnosis status across multiple sample sizes (*n*) and number of features (*p*) in the Simple Covariance Effect simulations. For each of 1,000 simulations, MANOVA is performed separately for site and diagnosis using Pillai's trace. The rejection rate across these simulations is reported as the percentage of *p*‐values less than .05. Scenarios where the rejection rate is less than 0.05 are colored in blue.

## Figures and Tables

**FIGURE A1:**
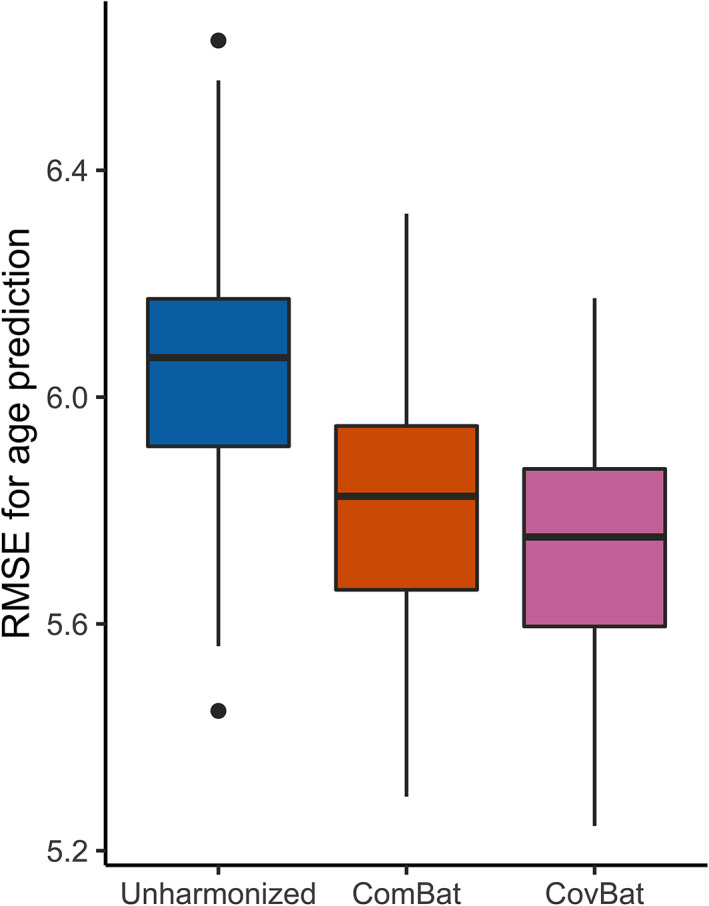
Multivariate pattern analysis experiments for detection of age using cortical thickness data. The data is randomly split into 50% training and 50% validation then used to train a random forests algorithm to predict age. RMSE values from 100 repetitions of this analysis are reported for unharmonized, ComBat‐adjusted, and CovBat‐adjusted data

**FIGURE A2:**
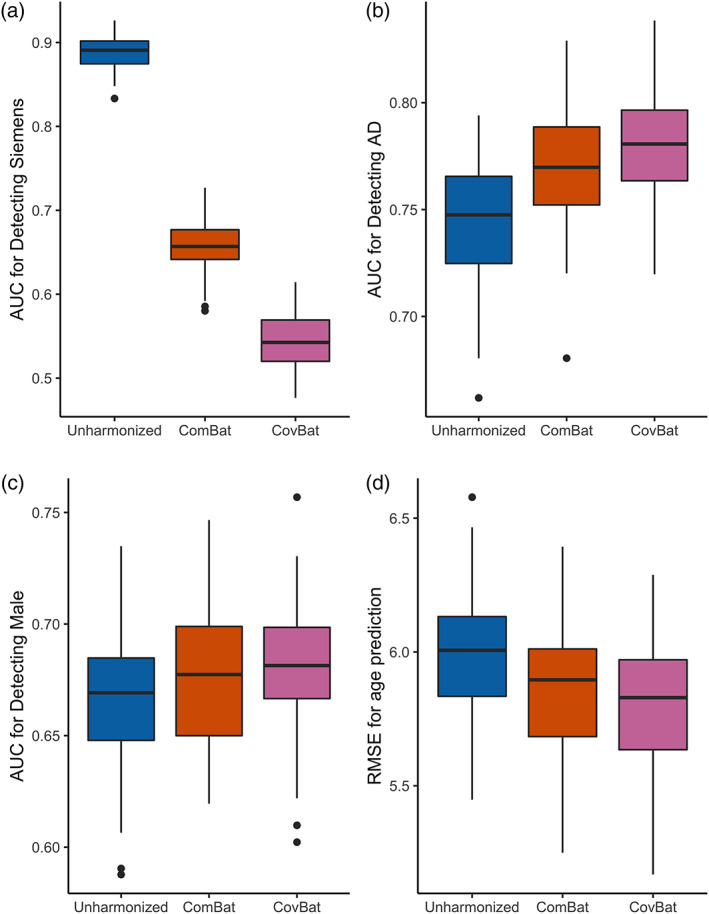
ML experiment results for harmonization using only training data. The data is randomly split into 270 training subjects and 235 testing subjects such that every site is represented in each group. The training set is then used to train a random forests algorithm to predict Siemens scanners or patient characteristics. a shows the AUC values for detection of Siemens. AUC values for detection of AD are displayed in b and detection of male in (c). RMSE values for prediction of age are displayed in (d)

## Data Availability

The data that support the findings of this study are available from the Alzheimer's Disease Neuroimaging Initiative. Restrictions apply to the availability of these data, which were used under license for this study. Data are available at adni.loni.usc.edu with the permission of ADNI.

## CovBat preserves prediction of age

ComBat has previously been shown to preserve age prediction via linear regression and support vector regression (Fortin et al., 2018). To evaluate whether this result holds in ADNI data for ComBat or CovBat, we propose an additional ML experiment. We (i) randomly split the subjects into 50% training set and 50% validation set, (ii) train a random forests algorithm to predict age, and (iii) assess predictive performance on the validation set via root‐mean‐square‐error (RMSE). We perform steps (i)–(iii) 100 times each for unharmonized, ComBat‐adjusted, and CovBat‐adjusted data. Figure A1 shows that the mean RMSE for age prediction decreases from 6.05 (±0.22) in unharmonized to 5.81 (±0.23) in ComBat‐adjusted data to 5.75 (±0.21) in CovBat‐adjusted data. These findings are consistent with previous work (Fortin et al., 2018) and show that CovBat provides similar recovery of the association between cortical thickness and age.

## Parameter estimation using training subset

Both ComBat and CovBat estimate and residualize out the covariate effects using the full data; however, there are cases were only a subset of the data is available when performing harmonization. For instance, if a group of subjects has already been acquired, prediction on subjects subsequently acquired on the same sites could only leverage data from the original sample. In this scenario, the new sample can be harmonized using ComBat or CovBat by estimating the covariate effect using the original sample, then proceeding with subsequent steps as usual.

We evaluate this modification by repeating our main ML analyses using ADNI data with different subsampling of the patients. Specifically, we replace step (i) in our ML experiments by instead splitting the sample into 270 training subjects and 235 testing subjects such that both the train and test sets contain at least one subject acquired at each site. We then apply ComBat and CovBat by estimating the covariate effects using only the training subjects. Figure A2 shows the results for all ML experiments. Detection of site (AUC 0.89±0.02 in raw data) still worsens after ComBat (0.66±0.03) and is almost at chance after CovBat (0.54±0.03). For detection of AD, improvements over unharmonized (AUC 0.74±0.03) are still demonstrated after ComBat adjustment (0.77±0.03) and CovBat adjustment (0.78±0.02). For detection of male, lesser improvement is observed from unharmonized (AUC 0.67±0.03) to ComBat (0.68±0.03) to CovBat (0.68±0.03). Mean RMSE for age prediction decreases from 5.99 (±0.22) in unharmonized to 5.86 (±0.23) in ComBat‐adjusted data to 5.82 (±0.22) in CovBat‐adjusted data. Overall, the results appear quite similar to harmonization using the full dataset, showing that CovBat performs well even when only a limited training subset is available. These results also demonstrate that splitting the sample across sites does not substantially mitigate site effects.
