# Supplementary material for: Mitigating site effects in covariance for machine learning in neuroimaging data
Source: Hum Brain Mapp. 2021 Dec 14;43(4):1179–95. doi: 10.1002/hbm.25688 (PMC8837590; doi:10.1002/hbm.25688)
Supplement: Supplementary file 1 — Figure S1 Covariance matrix for cortical thickness values acquired at the largest site. Site A is a Siemens Symphony 1.5T scanner. Regions of interest are labeled on the x‐axis and y‐axis. Figure S2: Simple Covariance Effect simulation AUCs for detection of site after applying CovBat including varying numbers of principal components. Each point represents the median and interquartile range of AUCs for detection of site across 1,000 simulations. The rank of the site covariance effect varies in each plot and the number of features is fixed at 62. Results are displayed for both 100 and 250 samples as separate lines. Figure S3: Diagnosis Affects Covariance simulation AUCs for detection of site after applying CovBat including varying numbers of principal components. Each point represents the median and interquartile range of AUCs for detection of site across 1,000 simulations. Differing sample sizes and numbers of features are represented by different lines in each plots. Figure S4: Diagnosis Affects Covariance simulation AUCs for detection of simulated diagnosis after applying CovBat including varying numbers of principal components. Each point represents the median and interquartile range of AUCs for detection of diagnosis across 1,000 simulations. Differing sample sizes and numbers of features are represented by different lines in each plots. Figure S5: AUC for machine learning detection after CovBat across varying numbers of PCs included in the model. For each number of PCs, the median and IQR for detection of either Siemens, male, or Alzheimer's disease are displayed. Each point represents 1,000 splits of the ADNI data into 50% training and 50% validation. Figure S6: DeLong's test p‐value Q–Q plots for comparing AUC between ComBat and CovBat. For each of the 100 train‐test splits within each MVPA experiment, DeLong's test is performed with the two‐sided null hypothesis that the AUC is different in ComBat‐adjusted and CovBat‐adjusted data. The p‐values for each exper [file HBM-43-1179-s001.pdf]

Mitigating Site Effects in Covariance for Machine Learning in Neuroimaging Data

Andrew A. Chen[a,b,*], Joanne C. Beer[a], Nicholas J. Tustison[c], Philip A. Cook[d], Russell T. Shinohara[a,b,1], Haochang Shou[a,b,1], and the Alzheimer's Disease Neuroimaging Initiative[e]

[a]Penn Statistics in Imaging and Visualization Center, Department of Biostatistics, Epidemiology, and Informatics, University of Pennsylvania, Philadelphia, PA 19104

[b]Center for Biomedical Image Computing and Analytics, University of Pennsylvania, Philadelphia, PA 19104

[c]Department of Radiology and Medical Imaging, University of Virginia, Irvine, Charlottesville, VA 22903

[d]Department of Radiology, University of Pennsylvania, Philadelphia, PA 19104

[e]Data used in preparation of this article were obtained from the Alzheimer's Disease Neuroimaging Initiative (ADNI) database (adni.loni.usc.edu). As such, the investigators within the ADNI contributed to the design and implementation of ADNI and/or provided data but did not participate in analysis or writing of this report. A complete listing of ADNI investigators can be found at: http://adni.loni.usc.edu/wp-content/uploads/how_to_apply/ADNI_Acknowledgement_List.pdf

[1]Equal contribution

***Correspondence: Andrew A. Chen**
andrewac@pennmedicine.upenn.edu

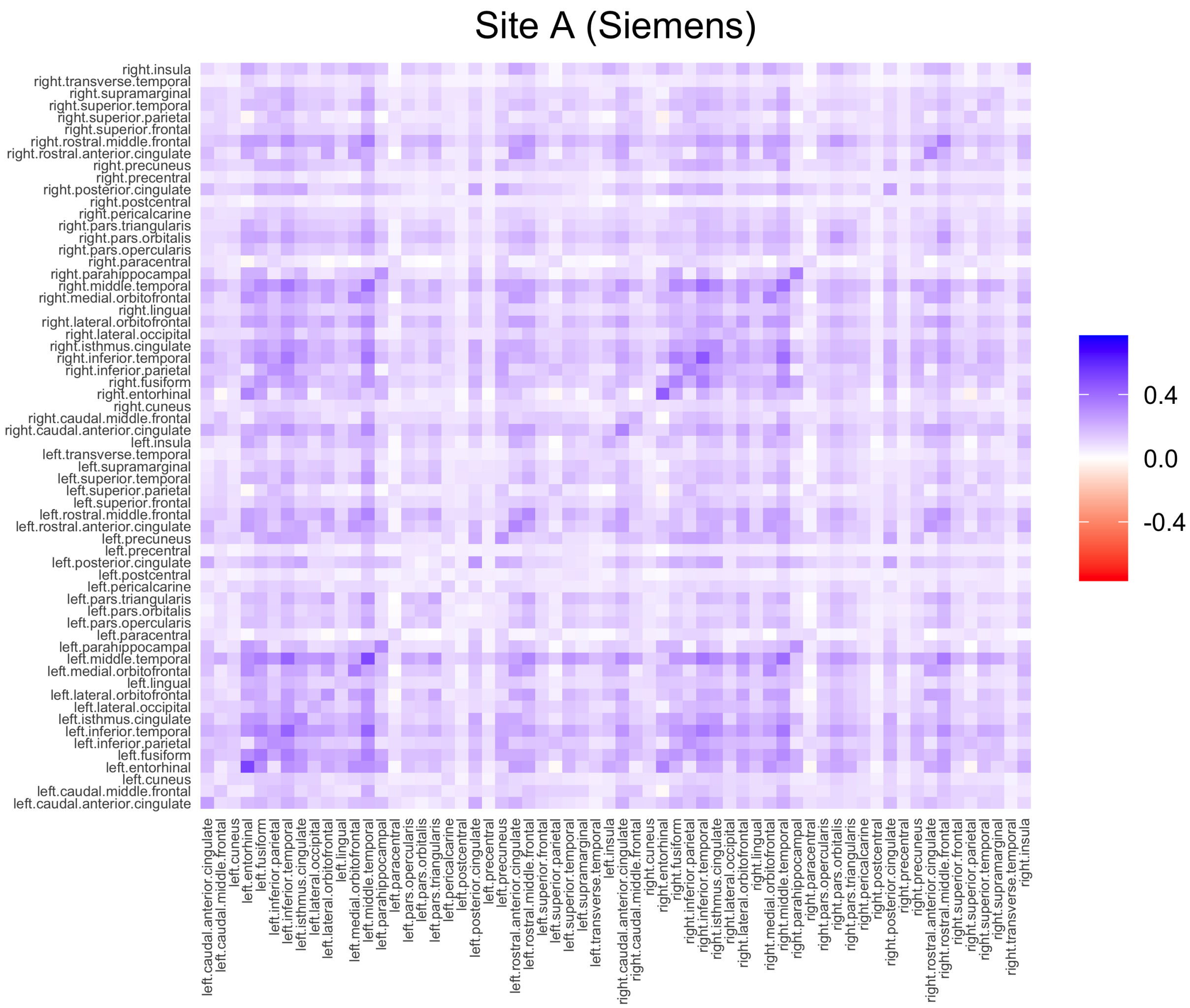

Supplementary Figure 1: **Covariance matrix for cortical thickness values acquired at the largest site.** Site A is a Siemens Symphony 1.5T scanner. Regions of interest are labelled on the x-axis and y-axis.

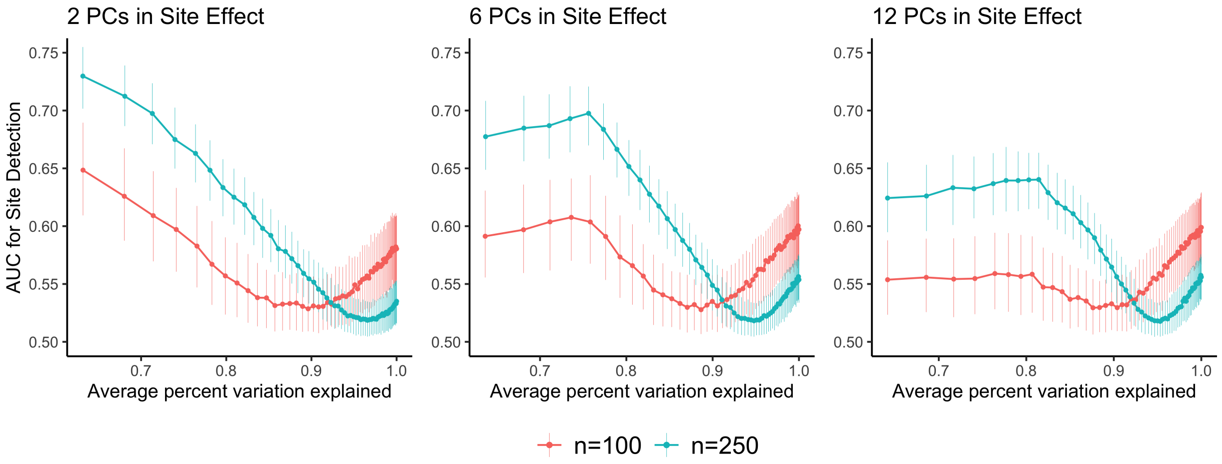

Supplementary Figure 2: **Simple Covariance Effect simulation AUCs for detection of site after applying CovBat including varying numbers of principal components.** Each point represents the median and interquartile range of AUCs for detection of site across 1000 simulations. The rank of the site covariance effect varies in each plot and the number of features is fixed at 62. Results are displayed for both 100 and 250 samples as separate lines.

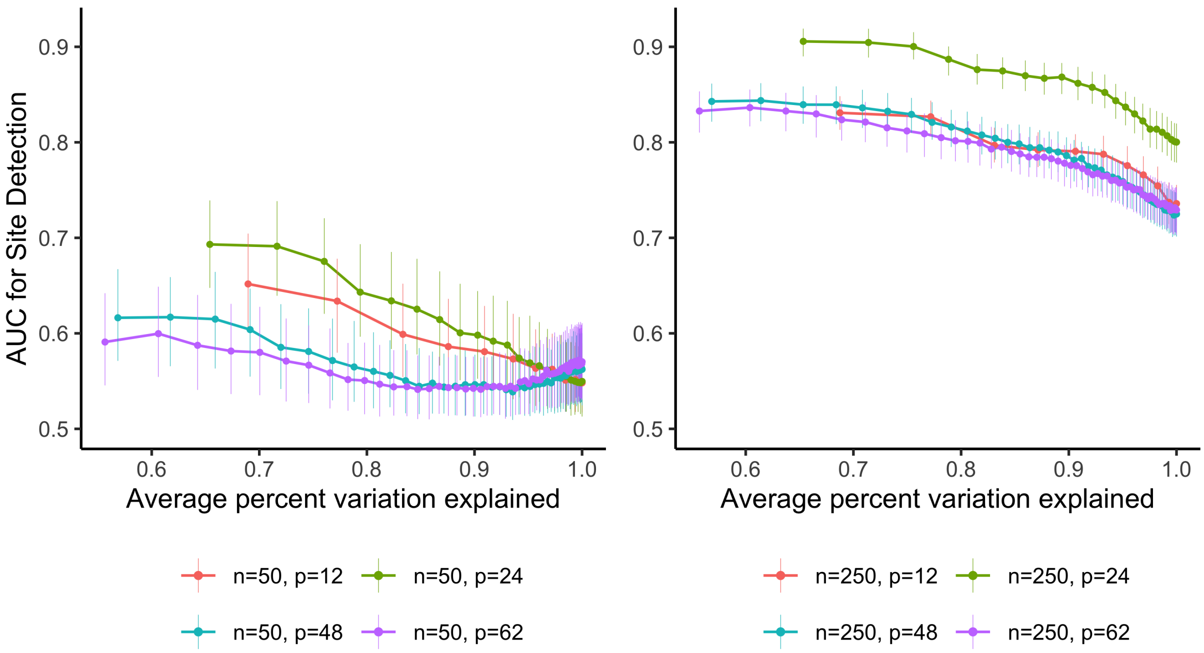

Supplementary Figure 3: **Diagnosis Affects Covariance simulation AUCs for detection of site after applying CovBat including varying numbers of principal components.** Each point represents the median and interquartile range of AUCs for detection of site across 1000 simulations. Differing sample sizes and numbers of features are represented by different lines in each plots.

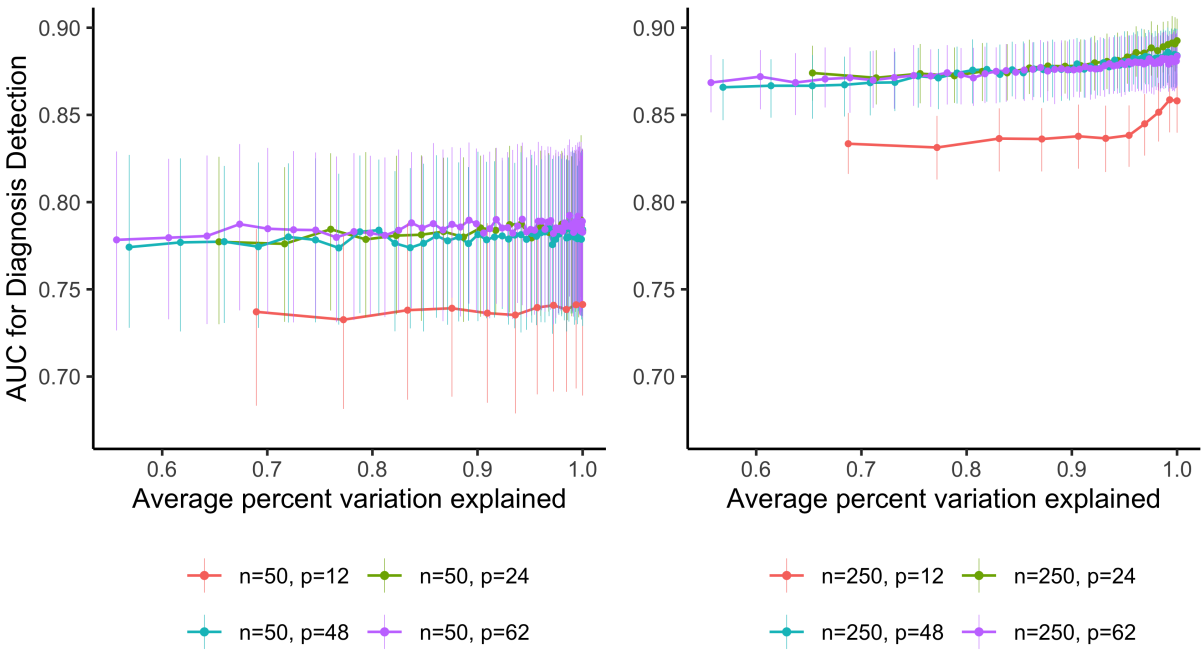

Supplementary Figure 4: **Diagnosis Affects Covariance simulation AUCs for detection of simulated diagnosis after applying CovBat including varying numbers of principal components.** Each point represents the median and interquartile range of AUCs for detection of diagnosis across 1000 simulations. Differing sample sizes and numbers of features are represented by different lines in each plots.

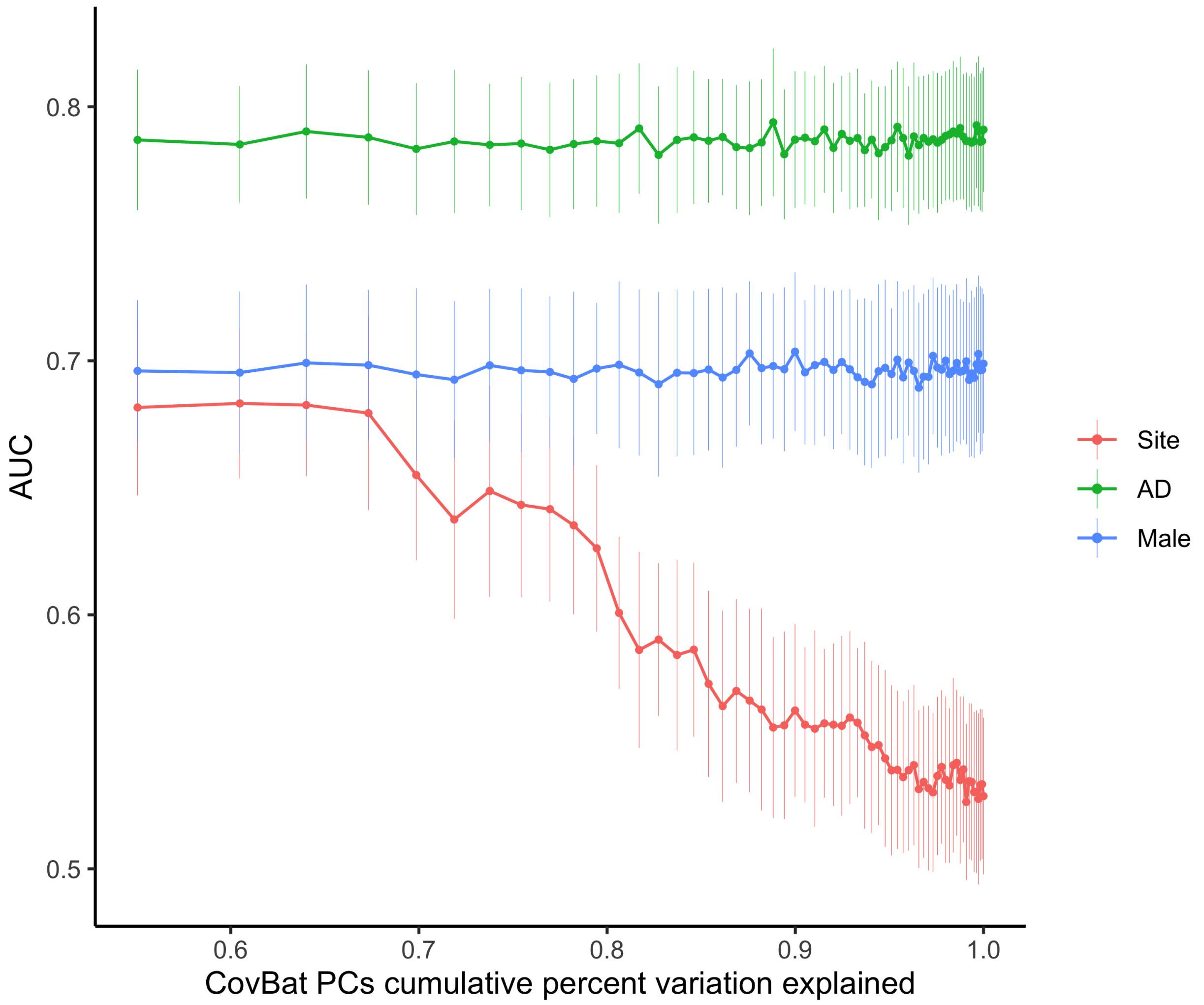

Supplementary Figure 5: **AUC for machine learning detection after CovBat across varying numbers of PCs included in the model.** For each number of PCs, the median and IQR for detection of either Siemens, male, or Alzheimer's disease are displayed. Each point represents 1000 splits of the ADNI data into 50% training and 50% validation.

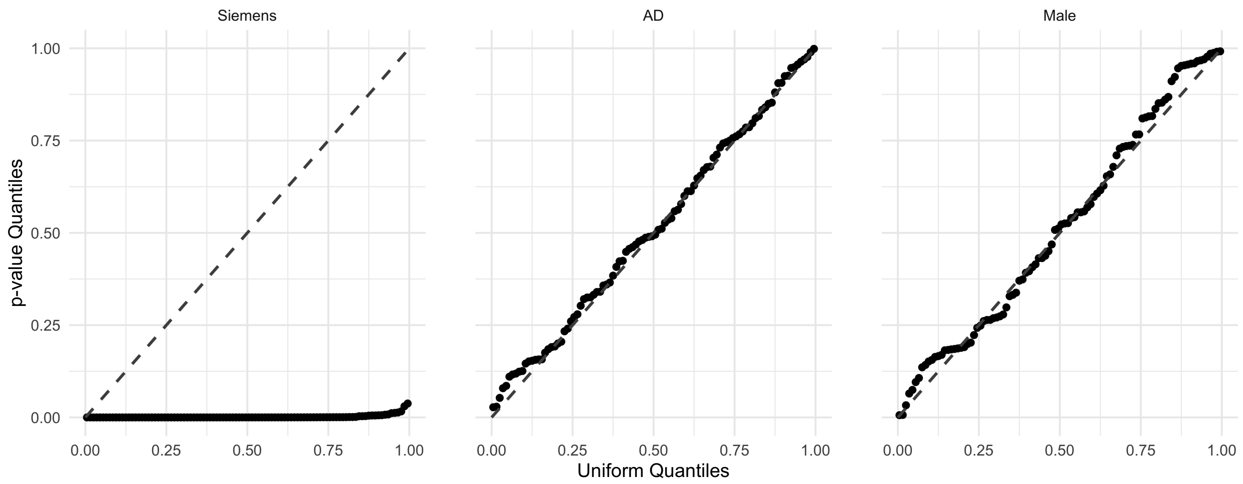

Supplementary Figure 6: **DeLong's test p-value Q-Q plots for comparing AUC between ComBat and CovBat.** For each of the 100 train-test splits within each MVPA experiment, DeLong's test is performed with the two-sided null hypothesis that the AUC is different in ComBat-adjusted and CovBat-adjusted data. The p-values for each experiment are compared to a uniform distribution from 0 to 1.

		ComBat Simulation			Diagnosis affects Mean		
n_i	p	Unharmonized	ComBat	CovBat	Unharmonized	ComBat	CovBat
25	12	0.61 (0.54-0.67)	0.58 (0.53-0.63)	0.62 (0.56-0.67)	0.69 (0.63-0.76)	0.58 (0.53-0.63)	0.57 (0.52-0.62)
	24	0.64 (0.57-0.71)	0.58 (0.53-0.63)	0.64 (0.58-0.69)	0.74 (0.68-0.80)	0.58 (0.53-0.65)	0.56 (0.52-0.61)
	48	0.67 (0.60-0.74)	0.58 (0.53-0.63)	0.66 (0.61-0.71)	0.72 (0.65-0.78)	0.56 (0.52-0.61)	0.62 (0.57-0.68)
	62	0.68 (0.61-0.75)	0.58 (0.53-0.63)	0.66 (0.61-0.72)	0.72 (0.65-0.79)	0.56 (0.52-0.61)	0.64 (0.58-0.70)
50	12	0.64 (0.58-0.70)	0.55 (0.52-0.59)	0.58 (0.53-0.62)	0.79 (0.74-0.83)	0.68 (0.62-0.73)	0.59 (0.55-0.64)
	24	0.69 (0.64-0.75)	0.55 (0.52-0.59)	0.59 (0.54-0.63)	0.84 (0.80-0.88)	0.72 (0.67-0.78)	0.59 (0.55-0.64)
	48	0.72 (0.67-0.78)	0.55 (0.52-0.59)	0.61 (0.57-0.65)	0.82 (0.77-0.86)	0.61 (0.56-0.67)	0.54 (0.51-0.57)
	62	0.74 (0.69-0.79)	0.55 (0.52-0.59)	0.61 (0.57-0.65)	0.81 (0.77-0.86)	0.58 (0.54-0.63)	0.55 (0.51-0.58)
100	12	0.67 (0.63-0.72)	0.54 (0.51-0.56)	0.55 (0.52-0.58)	0.86 (0.83-0.89)	0.78 (0.75-0.82)	0.71 (0.68-0.75)
	24	0.74 (0.70-0.78)	0.54 (0.51-0.56)	0.56 (0.53-0.59)	0.92 (0.90-0.94)	0.85 (0.82-0.88)	0.74 (0.71-0.78)
	48	0.79 (0.75-0.83)	0.54 (0.51-0.57)	0.57 (0.54-0.60)	0.90 (0.87-0.93)	0.75 (0.71-0.78)	0.62 (0.58-0.66)
	62	0.82 (0.78-0.85)	0.54 (0.51-0.57)	0.57 (0.54-0.60)	0.90 (0.87-0.93)	0.71 (0.67-0.76)	0.60 (0.57-0.64)
250	12	0.72 (0.67-0.75)	0.52 (0.51-0.54)	0.53 (0.51-0.55)	0.92 (0.91-0.94)	0.88 (0.87-0.90)	0.84 (0.83-0.86)
	24	0.80 (0.77-0.84)	0.52 (0.51-0.54)	0.53 (0.51-0.55)	0.97 (0.96-0.98)	0.95 (0.94-0.96)	0.90 (0.88-0.92)
	48	0.87 (0.84-0.89)	0.53 (0.51-0.55)	0.54 (0.52-0.56)	0.96 (0.95-0.97)	0.88 (0.87-0.90)	0.79 (0.77-0.81)
	62	0.89 (0.86-0.91)	0.53 (0.51-0.55)	0.54 (0.52-0.56)	0.97 (0.96-0.97)	0.87 (0.85-0.88)	0.79 (0.76-0.81)

Supplementary Table 1: **AUC results from MVPA simulations for detection of site across multiple sample sizes (n) and number of features (p) in the absence of predictor effects on covariance.** For each of 1000 simulations, the data is randomly split into 50% training and 50% validation then a random forests algorithm is trained using the training set to predict either Site 1 or the presence of the binary predictor. The median AUC across these simulations are reported with lower and upper quartiles displayed in parentheses. Scenarios where CovBat outperforms ComBat are colored in blue.

		Diagnosis affects Covariance			Covariance Only		
n_i	p	Unharmonized	ComBat	CovBat	Unharmonized	ComBat	CovBat
25	12	0.70 (0.63-0.77)	0.58 (0.53-0.63)	0.57 (0.53-0.62)	0.61 (0.56-0.68)	0.58 (0.53-0.63)	0.57 (0.52-0.62)
	24	0.74 (0.67-0.81)	0.58 (0.53-0.65)	0.57 (0.53-0.63)	0.64 (0.56-0.71)	0.57 (0.53-0.63)	0.58 (0.54-0.63)
	48	0.74 (0.66-0.80)	0.57 (0.52-0.61)	0.63 (0.57-0.69)	0.61 (0.55-0.67)	0.57 (0.52-0.62)	0.64 (0.60-0.70)
	62	0.73 (0.66-0.79)	0.57 (0.53-0.62)	0.65 (0.60-0.71)	0.60 (0.54-0.66)	0.57 (0.53-0.63)	0.65 (0.60-0.71)
50	12	0.78 (0.74-0.83)	0.67 (0.62-0.72)	0.56 (0.52-0.61)	0.70 (0.64-0.74)	0.66 (0.61-0.71)	0.56 (0.52-0.60)
	24	0.84 (0.79-0.88)	0.71 (0.65-0.77)	0.57 (0.53-0.61)	0.74 (0.69-0.79)	0.70 (0.65-0.75)	0.56 (0.52-0.60)
	48	0.82 (0.77-0.86)	0.62 (0.57-0.68)	0.55 (0.52-0.58)	0.67 (0.62-0.72)	0.60 (0.55-0.65)	0.55 (0.51-0.59)
	62	0.82 (0.78-0.87)	0.60 (0.55-0.65)	0.55 (0.52-0.59)	0.65 (0.60-0.70)	0.57 (0.53-0.62)	0.56 (0.52-0.60)
100	12	0.85 (0.82-0.88)	0.77 (0.73-0.80)	0.66 (0.63-0.70)	0.78 (0.75-0.81)	0.77 (0.73-0.80)	0.66 (0.62-0.69)
	24	0.91 (0.89-0.93)	0.84 (0.81-0.86)	0.69 (0.66-0.73)	0.84 (0.81-0.87)	0.83 (0.80-0.86)	0.69 (0.65-0.72)
	48	0.90 (0.87-0.93)	0.75 (0.71-0.79)	0.60 (0.56-0.63)	0.77 (0.74-0.80)	0.74 (0.70-0.78)	0.59 (0.55-0.62)
	62	0.90 (0.88-0.93)	0.72 (0.68-0.76)	0.59 (0.56-0.62)	0.75 (0.71-0.78)	0.71 (0.67-0.75)	0.58 (0.54-0.61)
250	12	0.90 (0.89-0.92)	0.85 (0.84-0.87)	0.78 (0.76-0.80)	0.85 (0.84-0.87)	0.85 (0.84-0.87)	0.77 (0.76-0.79)
	24	0.96 (0.95-0.97)	0.92 (0.91-0.93)	0.83 (0.82-0.85)	0.92 (0.91-0.93)	0.92 (0.91-0.93)	0.83 (0.81-0.85)
	48	0.96 (0.95-0.97)	0.87 (0.85-0.88)	0.76 (0.73-0.78)	0.87 (0.85-0.89)	0.87 (0.85-0.88)	0.75 (0.73-0.77)
	62	0.96 (0.95-0.97)	0.86 (0.84-0.87)	0.76 (0.73-0.78)	0.86 (0.84-0.88)	0.85 (0.83-0.87)	0.75 (0.73-0.78)

Supplementary Table 2: **AUC results from MVPA simulations for detection of site across multiple sample sizes (n) and number of features (p) where the predictor has effects on covariance.** For each of 1000 simulations, the data is randomly split into 50% training and 50% validation then a random forests algorithm is trained using the training set to predict either Site 1 or the presence of the binary predictor. The median AUC across these simulations are reported with lower and upper quartiles displayed in parentheses. Scenarios where CovBat outperforms ComBat are colored in blue.

		Diagnosis Affects Covariance			Covariance Only		
n	p	Unharmonized	ComBat	CovBat	Unharmonized	ComBat	CovBat
25	12	0.68 (0.60-0.75)	0.69 (0.61-0.76)	0.69 (0.61-0.76)	0.68 (0.61-0.75)	0.69 (0.61-0.76)	0.69 (0.61-0.77)
	24	0.71 (0.63-0.78)	0.72 (0.65-0.79)	0.73 (0.66-0.80)	0.72 (0.64-0.78)	0.73 (0.66-0.80)	0.73 (0.65-0.80)
	48	0.71 (0.64-0.78)	0.72 (0.65-0.79)	0.74 (0.66-0.80)	0.72 (0.65-0.79)	0.72 (0.65-0.79)	0.73 (0.66-0.80)
	62	0.72 (0.65-0.79)	0.73 (0.65-0.80)	0.74 (0.66-0.81)	0.72 (0.65-0.79)	0.73 (0.66-0.80)	0.74 (0.67-0.81)
50	12	0.73 (0.67-0.77)	0.73 (0.68-0.77)	0.74 (0.69-0.78)	0.73 (0.68-0.78)	0.73 (0.68-0.78)	0.74 (0.69-0.79)
	24	0.77 (0.72-0.81)	0.78 (0.72-0.82)	0.78 (0.73-0.83)	0.77 (0.72-0.82)	0.77 (0.73-0.82)	0.79 (0.73-0.83)
	48	0.76 (0.72-0.81)	0.77 (0.72-0.82)	0.78 (0.73-0.83)	0.77 (0.72-0.81)	0.77 (0.73-0.82)	0.78 (0.73-0.83)
	62	0.77 (0.72-0.82)	0.78 (0.73-0.83)	0.79 (0.73-0.83)	0.77 (0.72-0.82)	0.78 (0.73-0.82)	0.78 (0.73-0.83)
100	12	0.77 (0.74-0.80)	0.78 (0.74-0.81)	0.79 (0.75-0.82)	0.78 (0.75-0.81)	0.78 (0.74-0.81)	0.79 (0.75-0.82)
	24	0.82 (0.78-0.85)	0.82 (0.79-0.85)	0.84 (0.81-0.87)	0.82 (0.79-0.85)	0.82 (0.79-0.85)	0.84 (0.81-0.87)
	48	0.81 (0.78-0.84)	0.82 (0.79-0.85)	0.83 (0.80-0.86)	0.81 (0.78-0.84)	0.81 (0.78-0.84)	0.83 (0.80-0.86)
	62	0.82 (0.78-0.85)	0.82 (0.79-0.85)	0.83 (0.80-0.86)	0.82 (0.79-0.85)	0.82 (0.79-0.85)	0.83 (0.80-0.86)
250	12	0.83 (0.81-0.84)	0.83 (0.81-0.85)	0.84 (0.82-0.85)	0.83 (0.81-0.85)	0.83 (0.81-0.85)	0.84 (0.82-0.86)
	24	0.87 (0.85-0.88)	0.87 (0.85-0.88)	0.89 (0.87-0.90)	0.87 (0.85-0.88)	0.87 (0.85-0.88)	0.88 (0.87-0.90)
	48	0.86 (0.84-0.88)	0.86 (0.84-0.88)	0.88 (0.86-0.90)	0.86 (0.84-0.88)	0.86 (0.84-0.88)	0.88 (0.86-0.90)
	62	0.87 (0.85-0.88)	0.87 (0.85-0.88)	0.88 (0.86-0.90)	0.87 (0.85-0.88)	0.87 (0.85-0.88)	0.88 (0.86-0.89)

Supplementary Table 3: **AUC results from MVPA simulations for detection of simulated diagnosis across multiple sample sizes (n) and number of features (p) where the predictor has effects on covariance.** For each of 1000 simulations, the data is randomly split into 50% training and 50% validation then a random forests algorithm is trained using the training set to predict either Site 1 or the presence of the binary predictor. The median AUC across these simulations are reported with lower and upper quartiles displayed in parentheses.

n_i	p	Site			Diagnosis Status		
		Unharmonized (%)	ComBat (%)	CovBat (%)	Unharmonized (%)	ComBat (%)	CovBat (%)
25	12	99	41	16	98	99	99
	24	100	83	31	100	100	100
	48	100	76	8	99	99	99
	62	100	67	6	62	63	65
50	12	100	63	36	100	100	100
	24	100	95	61	100	100	100
	48	100	97	36	100	100	100
	62	100	99	40	100	100	100
100	12	100	63	40	100	100	100
	24	100	94	73	100	100	100
	48	100	98	62	100	100	100
	62	100	100	72	100	100	100
250	12	100	31	21	100	100	100
	24	100	70	48	100	100	100
	48	100	70	33	100	100	100
	62	100	88	50	100	100	100

Supplementary Table 4: **MANOVA rejection rates for associations with site and diagnosis status across multiple sample sizes (n) and number of features (p) in the Diagnosis affects Mean simulations.** For each of 1000 simulations, MANOVA is performed separately for site and diagnosis using Pillai's trace. The rejection rate across these simulations is reported as the percentage of p-values less than 0.05.

		Site			Diagnosis Status		
n_i	p	Unharmonized (%)	ComBat (%)	CovBat (%)	Unharmonized (%)	ComBat (%)	CovBat (%)
25	12	96	10	1	92	94	96
	24	100	44	4	100	100	100
	48	100	42	1	96	97	98
	62	100	40	1	62	64	66
50	12	100	14	2	100	100	100
	24	100	64	10	100	100	100
	48	100	73	3	100	100	100
	62	100	86	3	100	100	100
100	12	100	12	2	100	100	100
	24	100	60	11	100	100	100
	48	100	68	5	100	100	100
	62	100	87	9	100	100	100
250	12	100	2	0	100	100	100
	24	100	11	2	100	100	100
	48	100	8	0	100	100	100
	62	100	18	1	100	100	100

Supplementary Table 5: **MANOVA rejection rates for associations with site and diagnosis status across multiple sample sizes (n) and number of features (p) in the Diagnosis affects Covariance simulations.** For each of 1000 simulations, MANOVA is performed separately for site and diagnosis using Pillai's trace. The rejection rate across these simulations is reported as the percentage of p-values less than 0.05. Scenarios where the rejection rate is less than 0.05 are colored in blue.

		Site			Diagnosis Status		
n	p	Unharmonized (%)	ComBat (%)	CovBat (%)	Unharmonized (%)	ComBat (%)	CovBat (%)
25	12	94	1	0	74	78	82
	24	100	25	0	97	98	98
	36	100	48	1	99	99	99
	48	100	50	0	100	100	100
	62	100	47	1	96	97	98
50	12	100	1	0	99	99	99
	24	100	53	3	100	100	100
	36	100	83	6	100	100	100
	48	100	92	9	100	100	100
	62	100	99	16	100	100	100
100	12	100	2	0	100	100	100
	24	100	52	7	100	100	100
	36	100	86	18	100	100	100
	48	100	95	27	100	100	100
	62	100	100	61	100	100	100
250	12	100	0	0	100	100	100
	24	100	9	1	100	100	100
	36	100	29	6	100	100	100
	48	100	48	8	100	100	100
	62	100	83	29	100	100	100
500	12	100	0	0	100	100	100
	24	100	0	0	100	100	100
	36	100	0	0	100	100	100
	48	100	0	0	100	100	100
	62	100	4	0	100	100	100

Supplementary Table 6: **MANOVA rejection rates for associations with site and diagnosis status across multiple sample sizes (n) and number of features (p) in the Simple Covariance Effect simulations.** For each of 1000 simulations, MANOVA is performed separately for site and diagnosis using Pillai's trace. The rejection rate across these simulations is reported as the percentage of p-values less than 0.05. Scenarios where the rejection rate is less than 0.05 are colored in blue.
